# ER stress decreases exosome production through adiponectin/T-cadherin-dependent and -independent pathways

**DOI:** 10.1016/j.jbc.2023.105114

**Published:** 2023-07-29

**Authors:** Keita Fukuoka, Ryohei Mineo, Shunbun Kita, Shiro Fukuda, Tomonori Okita, Emi Kawada-Horitani, Masahito Iioka, Kohei Fujii, Keitaro Kawada, Yuya Fujishima, Hitoshi Nishizawa, Norikazu Maeda, Iichiro Shimomura

**Affiliations:** 1 Department of Metabolic Medicine, Graduate School of Medicine, Osaka University, Osaka, Japan; 2 Department of Adipose Management, Graduate School of Medicine, Osaka University, Osaka, Japan; 3 Department of Metabolism and Atherosclerosis, Graduate School of Medicine, Osaka University, Osaka, Japan

**Keywords:** ER stress, exosome, extracellular vesicle, adiponectin, T-cadherin

## Abstract

Exosomes, extracellular vesicles (EVs) produced within cells, mediate both the disposal of intracellular waste and communication with distant cells, and they are involved in a variety of disease processes. Although disease modifications of exosome cargos have been well studied, it has been poorly investigated how disease processes, such as endoplasmic reticulum (ER) stress, affect EV production. We previously reported that adiponectin, an adipocyte-secreted salutary factor, increases systemic exosome levels through T-cadherin-mediated enhancement of exosome biogenesis. In the present study, we demonstrated that adiponectin/T-cadherin-dependent EV production was susceptible to ER stress and that low-dose tunicamycin significantly reduced EV production in the presence, but not in the absence, of adiponectin. Moreover, pharmacological or genetic activation of inositol-requiring enzyme 1α, a central regulator of ER stress, downregulated T-cadherin at the mRNA and protein levels as well as attenuated EV production. In addition, adiponectin/T-cadherin-independent EV production was attenuated under ER stress conditions. Repeated administration of tunicamycin to mice decreased circulating small EVs without decreasing tissue T-cadherin expression. Mechanistically, inositol-requiring enzyme 1α activation by silencing of the X-box binding protein 1 transcription factor upregulated the canonical interferon pathway and decreased EV production. The interferon pathway, when it was activated by polyinosinic–polycytidylic acid, also significantly attenuated EV production. Thus, we concluded that ER stress decreases exosome production through adiponectin/T-cadherin-dependent and -independent pathways.

Small vesicles delimited by a lipid bilayer released from cells are defined as extracellular vesicles (EVs) (1). Based on their generating mechanism, EVs can be defined in subpopulations as “ectosomes” that are shed directly from the plasma membrane and “exosomes” that are released by exocytosis of multivesicular bodies, in which the limiting membrane of multivesicular bodies buds inward to generate exosomes (intraluminal vesicles) in the internal space of endosomes (2). Importantly, exosomes are considered to be secreted from virtually all cells, if not all, in any tissue or organ under various conditions. Thus, exosomes have been shown to mediate diverse cellular communications from homeostatic to disease-exacerbating roles.

Cells can be subjected to a variety of stress conditions called cellular stresses. Among these, endoplasmic reticulum (ER) stress is thought to govern cell metabolism, function, and even fate. ER stress occurs when protein folding exceeds the ability of the ER to fold, which activates three unfolded protein sensors, namely, inositol-requiring enzyme 1α (IRE1α), protein kinase RNA-like ER kinase, and activating transcription factor 6α. These sensors are conserved among mammals, and signaling by these sensors upregulates the transcription of genes encoding ER chaperones, oxidoreductases, and ER-associated degradation components (3). IRE1α is an ER transmembrane protein containing two enzymatic activities, namely, a kinase and an endoribonuclease (RNase), both residing on its cytosolic face (4). IRE1α senses ER unfolded proteins through its ER luminal domain, which becomes oligomerized and autophosphorylated during ER stress (5, 6, 7). Phosphorylated IRE1α cleaves X-box binding protein 1 (*XBP1*) mRNA at specific sites to generate a mature mRNA encoding an active transcription factor, XBP1s, which directly binds to the promoter region of ER chaperone genes (8, 9, 10). In addition, hyperactivated IRE1α has been shown to target multiple mRNAs for degradation upon stress in an XBP1-independent manner, which is known as regulated IRE1-dependent mRNA decay. Regulated IRE1-dependent mRNA decay substrates are downregulated following dosing with tunicamycin (TM) or in the presence of hyperactivated IRE1α because of X*bp*1 depletion in mice (11, 12).

Adiponectin is a circulating factor produced by adipocytes. Adiponectin is assembled intracellularly and secreted as a trimer, hexamer, or high molecular weight multimer. Clinically, hexameric and multimeric adiponectins bind to T-cadherin, a unique cadherin with a glycosylphosphatidylinositol anchor at its C terminus instead of transmembrane and cytoplasmic domains, thereby promoting exosome biosynthesis and secretion (13, 14, 15). Such enhanced exosome production by adiponectin has been observed in endothelial, skeletal muscle, and mesenchymal stem/stromal cells and is considered to account for various organ protections by adiponectin, including muscle regeneration and recovery from acute renal ischemia‒reperfusion injury (16, 17, 18, 19). Despite its importance, the molecular regulation of T-cadherin has been poorly explored, except for methylation-induced downregulation in cancer metastasis (20).

In the present study, we found that T-cadherin was downregulated by ER stress through IRE1α activation at the mRNA and protein levels. ER stress also decreased EV production through adiponectin/T-cadherin-dependent and -independent pathways as well as reduced plasma EV levels in mice. Interferon (IFN) pathway activation may account for ER stress–induced suppression of EV production.

## Results

### Pharmacological induction of ER stress reduces both T-cadherin protein and mRNA levels in multiple cell lines

**Figure 1:**
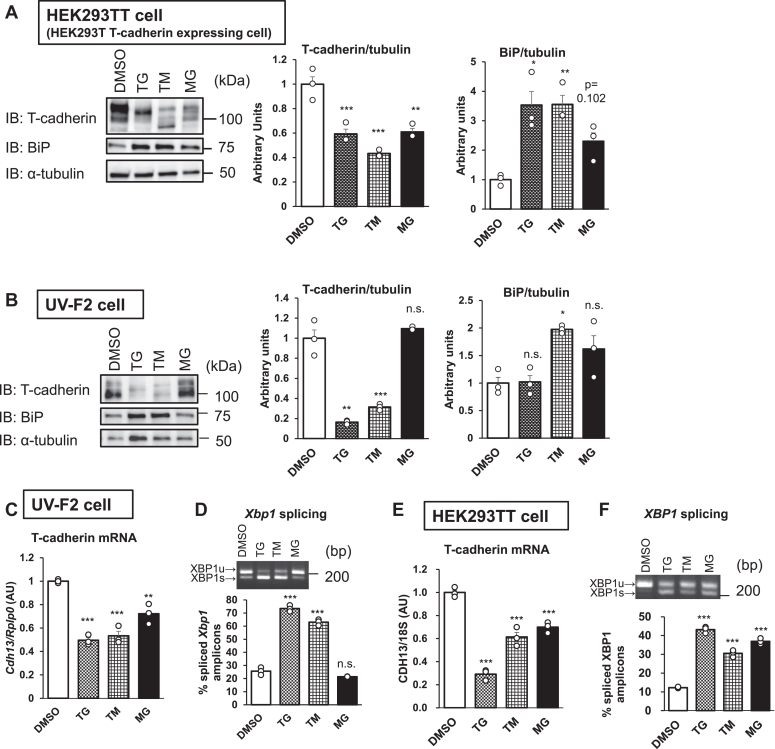
**Pharmacological induction of ER stress reduces T-cadherin protein and mRNA levels in cells.***A*, HEK293T cells stably expressing T-cadherin (293TT cells) were treated with 3 μmol/l thapsigargin (TG), 1 μmol/l tunicamycin (TM), or 1 μmol/l MG-132 (MG) for 24 h. T-cadherin and GRP78/BiP expression as normalized to α-tubulin. Whole-cell lysates (n = 3). *B*, UV-F2 cells treated with 0.3 μmol/l TG, 3 μmol/l TM, or 1 μmol/l MG for 24 h. Whole-cell lysates (n = 3). T-cadherin and BiP expression as normalized to α-tubulin. *C* and *D*, UV-F2 cells treated with 0.3 μmol/l TG, 3 μmol/l TM, or 1 μmol/l MG for 24 h. Whole-cell lysates (n = 3). *C*, T-cadherin mRNA expression as normalized to *Rplp0*. *D*, splicing rates of *Xbp1* amplicons analyzed by polyacrylamide gel electrophoresis. *E* and *F*, 293TT cells treated with 3 μmol/l TG, 1 μmol/l TM, or 1 μmol/l MG for 24 h. *E*, T-cadherin mRNA expression as normalized to *Rplp0*. *F*, splicing rates of *XBP1* amplicons analyzed by polyacrylamide gel electrophoresis. Data are presented as the mean ± SEM. The results expressed are representatives of two separate experiments. For *A*–*F*, statistical analyses were performed using one-way analysis of variance with Dunnett’s multiple comparisons. ∗*p* < 0.05, ∗∗*p* < 0.01, and ∗∗∗*p* < 0.001. BiP, binding protein; ER, endoplasmic reticulum; GRP78, glucose-regulated protein 78; XBP1, X-box binding protein 1; HEK293T, human embryonic kidney 293T cell line.

We examined T-cadherin protein expression in human embryonic kidney 293T (HEK293T) cells stably expressing T-cadherin (HEK293TT, Fig. S1*A*). Cells were treated with thapsigargin (TG), TM, or MG132 (MG) for 24 h, which cause ER stress *via* different mechanisms. Western blot analysis of whole-cell lysates showed that all compounds reduced T-cadherin protein levels and that they increased the expression of the glucose-regulated protein, glucose-regulated protein 78/binding protein (BiP), a well-known ER stress marker (Fig. 1*A*). Similarly, TG or TM reduced T-cadherin protein levels in UV-F2 endothelial cells and Chinese hamster ovary (CHO) cells stably expressing T-cadherin (CHO-T cell) (Figs. 1*B* and S1, *A* and *B*), but MG did not effectively reduce T-cadherin protein or induce BiP in these cells. These data suggested that T-cadherin protein reduction under ER stress is not a cell type–specific phenomenon.

To further investigate the mechanism of T-cadherin protein reduction under ER stress conditions, we examined T-cadherin mRNA expression in UV-F2 cells and found that the ER stress–inducing compounds decreased T-cadherin mRNA expression but increased *Xbp1* mRNA splicing (Fig. 1, *C* and *D*). Interestingly, a dose-dependent reduction in T-cadherin mRNA expression was also observed in CHO-T cells, which stably express T-cadherin complementary DNA (cDNA) driven by a retrovirus transcription element and otherwise have no detectable endogenous T-cadherin expression (Fig. S1, *C*–*E*) (13). These findings suggested that T-cadherin mRNA is downregulated because of reduced stability of the transcribed mRNA of T-cadherin under ER stress conditions. Similarly, the ER stress–inducing compounds decreased T-cadherin mRNA expression but increased *XBP1* mRNA splicing in HEK293TT cells (Fig. 1, *E* and *F*). The time-course experiment using HEK293TT cells demonstrated that T-cadherin mRNA was not downregulated at 2 and 4 h after TM treatment but was downregulated at 10 and 24 h after TM treatment (Fig. S1*F*). In contrast, *XBP1* splicing was elevated at 2 and 4 h but elevated to a lesser extent at 10 and 24 h (Fig. S1*G*).

### T-cadherin mRNA downregulation during ER stress is dependent on IRE1α

**Figure 2:**
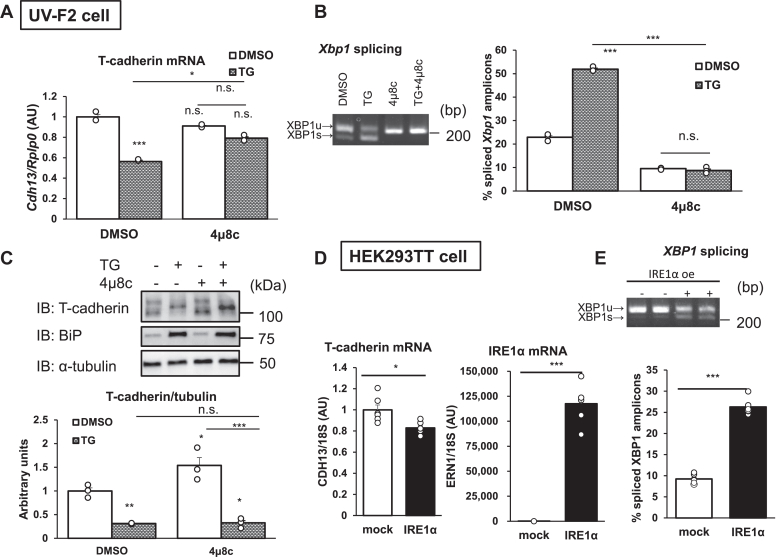
**T-cadherin mRNA downregulation under ER stress is IRE1α dependent.***A*–*C*, UV-F2 cells treated with 0.3 μmol/l TG and 30 μmol/l 4μ8c for 24 h. *A*, T-cadherin mRNA expression as normalized to *Rplp0* (n = 3). *B*, splicing rates of *Xbp1* amplicons analyzed by polyacrylamide gel electrophoresis. *C*, representative Western blots of whole-cell lysates (n = 3). T-cadherin expression as normalized to α-tubulin. *D* and *E*, transient overexpression of IRE1α in 293TT cells. *D*, T-cadherin and IRE1α mRNA expression as normalized to *Rplp0* (n = 6). *E*, splicing rates of *XBP1* amplicons analyzed by polyacrylamide gel electrophoresis. Data are presented as the mean ± SEM. The results expressed are representatives of two separate experiments. For *A*–*C*, statistical analyses were performed using one-way analysis of variance with Tukey’s multiple comparisons. *D* and *E*, statistical analyses were performed using Student’s *t* test. ∗*p* < 0.05, ∗∗*p* < 0.0, and ∗∗∗*p* < 0.001. ER, endoplasmic reticulum; IRE1α, inositol-requiring enzyme 1α; TG, thapsigargin; XBP1, X-box binding protein 1.

IRE1α is activated by ER stress and catalyzes the degradation of multiple mRNAs in an XBP1-independent manner (21). We found a candidate IRE1α recognition site in the T-cadherin mRNA sequence (Fig. S2). Therefore, we hypothesized that T-cadherin mRNA is degraded through IRE1α ribonuclease activity under ER stress conditions. To test this hypothesis, we investigated whether the selective IRE1 RNase inhibitor, 4μ8c, inhibits T-cadherin mRNA reduction under ER stress conditions in UV-F2 cells. TG treatment reduced T-cadherin mRNA expression, and this mRNA reduction was significantly attenuated by 4μ8c cotreatment (Fig. 2*A*). Similarly, XBP1 splicing was upregulated by TG treatment but was completely blocked by 4μ8c cotreatment (Fig. 2*B*). These results suggested that IRE1α ribonuclease activity is, at least in part, responsible for T-cadherin mRNA decay under ER stress conditions. Interestingly, the reduction in T-cadherin protein expression by TG treatment was not rescued by 4μ8c cotreatment (Fig. 2*C*). Therefore, another pathway may also attenuate T-cadherin protein expression under ER stress conditions.

To further verify T-cadherin mRNA downregulation through IRE1α ribonuclease activity, we transiently overexpressed IRE1α, which is self-activated by clustering of overproduced IRE1α proteins (22). As expected, IRE1α overexpression in HEK293TT cells reduced T-cadherin mRNA expression (Fig. 2*D*), in accordance with the increased *XBP1* splicing, which suggested that overexpression of IRE1α induced its RNase activity and reduced T-cadherin mRNA levels (Fig. 2*E*).

### Pharmacological induction of ER stress reduces EV production from cells through adiponectin/T-cadherin-dependent and -independent pathways

**Figure 3:**
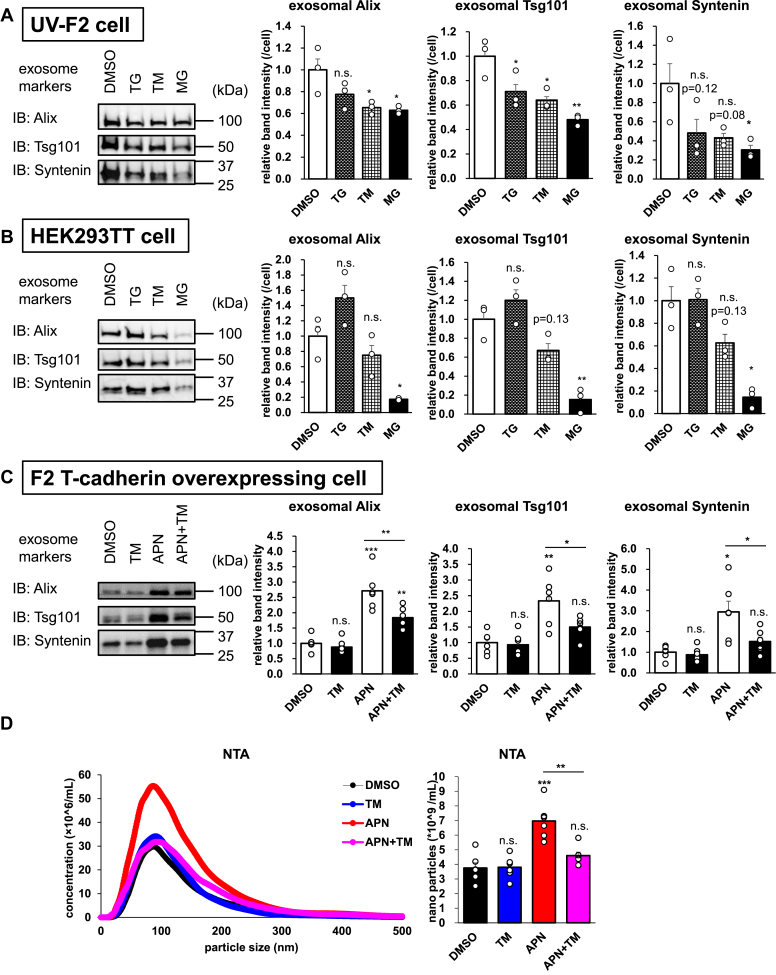
**Pharmacological induction of ER stress reduces EV production from cells.***A*–*C*, Western blots of EV fractions from the conditioned media. *A*, UV-F2 cells treated with 0.3 μmol/l TG, 3 μmol/l TM, or 1 μmol/l MG for 24 h (n = 3). *B*, 293TT cells treated with 3 μmol/l TG, 1 μmol/l TM, or 1 μmol/l MG for 24 h (n = 3). *C* and *D*, F2 T-cadherin stably expressing cells cultured with 5% exosome-free adiponectin KO mouse serum with or without 100 nmol/l TM and 20 μg/ml adiponectin for 48 h (n = 6). *C*, Western blots of EV fractions from the conditioned media. *D*, nanotracking analysis of the conditioned media. Data are presented as the mean ± SEM. The results expressed are representatives of two separate experiments. For *A* and *B*, statistical analyses were performed using one-way analysis of variance with Dunnett’s multiple comparisons. For *C* and *D*, statistical analyses were performed using one-way analysis of variance with Tukey’s multiple comparisons. ∗*p* < 0.05, ∗∗*p* < 0.01, and ∗∗∗*p* < 0.001. ER, endoplasmic reticulum; EV, extracellular vesicle; MG, MG132; TG, thapsigargin; TM, tunicamycin.

We have previously reported that T-cadherin binds adiponectin and mediates exosome production by adiponectin (14). Because ER stress attenuated T-cadherin expression, we investigated if the cells produce fewer exosomes under ER stress than under normal conditions. When we treated UV-F2 cells with TG, TM, and MG, the typical exosome markers in semipurified EV fractions were significantly reduced by these agents (Fig. 3*A*). Similarly, HEK293TT cells showed lower exosome marker expression when treated with TM or MG but not with TG (Figs. 3*B* and S3*A*). TG, a sarco/endoplasmic reticulum calcium-ATPase inhibitor, causes ER stress by increasing the intracellular calcium concentration (23), and increased intracellular calcium enhances exosome secretion (24). Therefore, the number of produced exosomes may depend on the balance between the decrease in exosome synthesis under ER stress and the increase in exosome secretion because of the increased intracellular calcium in the case of TG. The time-course study of EV production demonstrated a linear increase in exosome markers in the EV fraction purified from the conditioned media and its suppression by TM (Fig. S3*A*). A reduction in EV production was also observed in CHO-T cells (Fig. S3, *B*–*D*). Such decreased EV production under ER stress conditions was not recovered by 4μ8c cotreatment (Fig. S3*E*). We next evaluated whether ER stress abolishes the effect of adiponectin on EV production. As we have previously reported (14), adiponectin increased exosome production from F2T cells when the secreted exosomes were measured with exosome-specific marker protein expression by Western blot analysis and with exosome-sized particle counts by nanoparticle tracking analysis (Fig. 3, *C* and *D*). The low-dose TM treatment decreased EV production in the presence of adiponectin, but it did not affect EV production in the absence of adiponectin (Fig. 3, *C* and *D*). The concentration of TM in this experiment was low to avoid cytotoxicity during the 48 h incubation period, and T-cadherin expression was not reduced under these conditions at either the protein or mRNA level (Fig. S3, *F* and *G*). However, a higher dose of TM significantly decreased T-cadherin expression after 24 h of incubation (Fig. 1). There was no significant cytotoxicity observed as assessed by cell yields (Fig. S3*H*), and the levels of exosome markers were normalized by cell yields (Fig. 3, *A* and *B*). These findings suggested that cells produce fewer exosomes under ER stress conditions independent of IRE1α activation and that adiponectin/T-cadherin-mediated exosome production is highly sensitive to ER stress.

### IRE1α activates the IFN pathway, which downregulates T-cadherin expression and EV production

**Figure 4:**
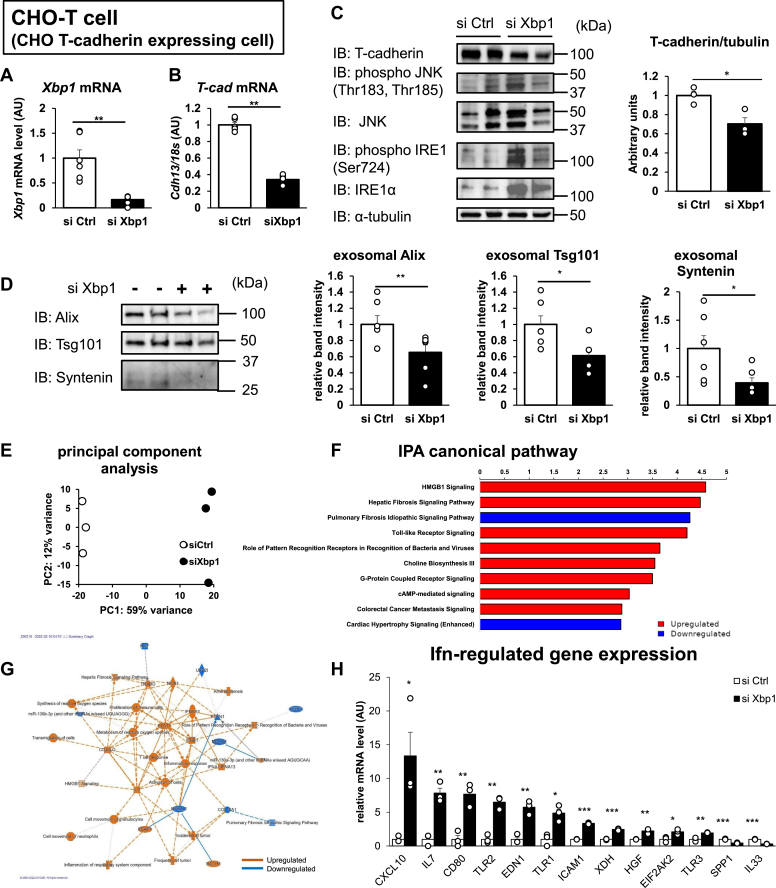
**The IFN pathway is associated with ER stress–induced downregulation of EV production.***A*–*H*, CHO T-cadherin stably expressing cells (CHO-T cells) transfected with the indicated siRNA for 48 h. *A*, *Xbp1* expression as normalized to total RNA (n = 6). *B*, T-cadherin mRNA expression as normalized to *Rplp0* (n = 6). *C*, Western blots of whole-cell lysates (n = 3). *D*, Western blots of EV fractions from the conditioned media. *E*, principal component analysis (n = 3). *F*–*H*, pathway analysis with ingenuity pathway analysis (IPA). *F*, the canonical pathway in differentially expressed genes. *G*, graphic summary of the canonical pathway. *H*, IFN-regulated gene expression. Data are presented as the mean ± SEM. The results expressed are representatives of two separate experiments. *A*–*D* and *H*, statistical analyses were performed using one-way analysis of variance with Student’s *t* test. ∗*p* < 0.05, ∗∗*p* < 0.01, and ∗∗∗*p* < 0.001. CHO, Chinese hamster ovary cell line; ER, endoplasmic reticulum; EV, extracellular vesicle; IFN, interferon; Xbp1, X-box binding protein 1.

Nicholas *et al.* (25) reported that *XBP1* knockdown activates IRE1α. In the present study, *Xbp1* silencing (85% knockdown) in CHO-T cells reduced T-cadherin gene expression to 65% (Figs. 4, *A* and *B*, and S4*A*), and T-cadherin protein expression was also significantly reduced by *Xbp1* knockdown (Fig. 4*C*). *Xbp1* silencing increased phosphorylated c-Jun N-terminal kinase and IRE1α, which confirmed that XBP1 depletion induced IRE1α activation (Fig. 4*C*). EV production was also decreased by *Xbp1* silencing (Fig. 4*D*). Next, RNA-Seq was performed to elucidate the biological pathway that limits EV production by IRE1α activation. In total, there were 457 and 403 genes significantly upregulated and downregulated by *Xbp1* silencing, respectively (Table S1). Principal component analysis indicated that *Xbp1* knockdown changed the gene expression profiles (Fig. 4*E*). The T-cadherin gene was one of the most downregulated genes by *Xbp1* silencing (Fig. S4*B*). Although we hypothesized the downregulation of multiple genes that are reported to be necessary for exosome formation or secretion, we only found one gene, namely, T-cadherin (Table S2). Ingenuity pathway analysis revealed that *Xbp1* silencing resulted in the upregulation of innate immune responses, such as signal transducer and activator of transcription 1, interferon alpha and beta receptor subunit 1, and inflammatory response pathways (Fig. 4, *F* and *G*). Many IFN-regulated genes were upregulated by *Xbp1* silencing (Fig. 4*H*). Carolina *et al.* (26) reported that IFN-induced ISGylation of TSG101 inhibits exosome production, and Maja *et al.* (27) also reported that *Ifnb1* gene expression is upregulated by IRE1α RNase activity in the BV-2 microglia-like cell line. Therefore, IFN-regulated pathway activation by IRE1α activation may play a role for the attenuated exosome production by Xbp1 silencing observed in the present study. To test this hypothesis, we treated UV-F2 cells with polyinosinic–polycytidylic acid [poly(I:C)]. As expected, poly(I:C) increased the expression of known IFN-regulated genes, such as protein kinase R and Toll-like receptor 3, and it decreased T-cadherin mRNA expression (Fig. S5*A*) and exosome markers in EV fractions (Fig. S5*B*). Importantly, poly(I:C) neither elevated *Xbp1* splicing (Fig. S5*C*) nor induced glucose-regulated protein 78 mRNA (Fig. S5*A*), which suggested that poly(I:C) did not activate any ER stress pathway. These data suggested that activation of IRE1α may be sufficient but not necessary to downregulate T-cadherin mRNA and exosome production.

### TM-induced ER stress reduces plasma EV *in vivo*

**Figure 5:**
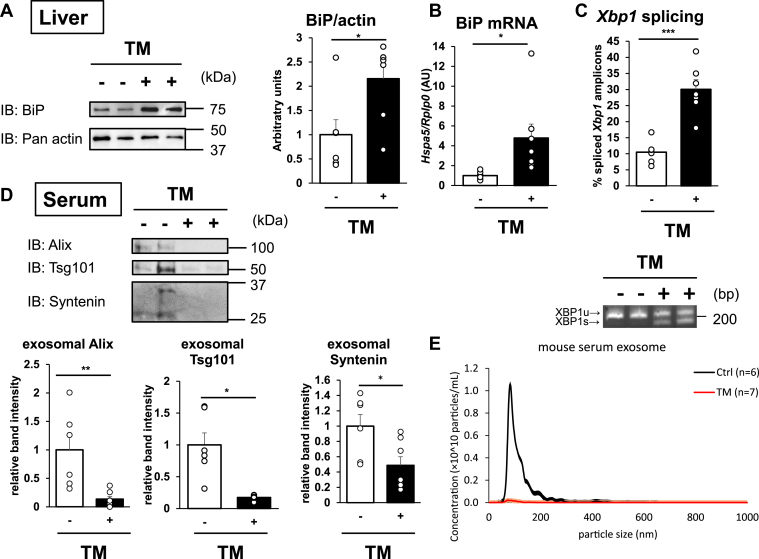
**Tunicamycin (TM)-induced ER stress reduces plasma small EVs in mice.** Male C57BL6/J mice were challenged with TM (1 μg/g body weight/day) or vehicle control for 2 days (n = 6–7). *A*, liver tissue lysates subjected to Western blot analysis with the indicated antibodies. *B*, BiP mRNA expression in the liver as normalized to *Rplp0*. *C*, splicing rates of *Xbp1* amplicons in liver RNA as analyzed by polyacrylamide gel electrophoresis. *D*, Western blots of exosome markers in the EV fractions from the plasma. *E*, nanotracking analysis (NTA) of purified plasma EV fractions. Data are presented as the mean ± SEM. The results expressed are representative of two separate experiments. Statistical analyses were performed using one-way analysis of variance with Student’s *t* test. ∗*p* < 0.05, ∗∗*p* < 0.01, and ∗∗∗*p* < 0.001. BiP, binding protein; ER, endoplasmic reticulum; EV, extracellular vesicle; Xbp1, X-box binding protein 1.

We next tested whether ER stress reduces EV production *in vivo*. Male C57BL6/J mice were challenged with i.p. TM (1 μg/g body weight/day) or vehicle for 2 days, which demonstrated that body weight was slightly reduced by TM injection (Fig. S6*A*). As reported previously (28, 29), upregulation of BiP at both the mRNA and protein levels as well as an increase in *Xbp1* splicing was observed in the livers of TM-injected mice, which confirmed that TM induced ER stress (Fig. 5, *A*–*C*). However, ER stress was not induced in the aorta, heart, and muscle where T-cadherin is highly expressed (Fig. S6, *B*–*D*) (30). We then analyzed the amounts of plasma EVs. As expected, the exosome markers of semipurified EV fractions were significantly reduced by TM treatment (Fig. 5*D*). Nanoparticle tracking analysis of the purified small EV fractions indicated that TM treatment reduced the number of exosome-sized particles in the plasma (Figs. 5*E* and S6*E*). The decrease of circulating small EVs in the plasma was independent of body weight reduction. Under fasting conditions, TM injection reduced EVs without significant changes in body weight (Fig. S7).

## Discussion

In the present study, we showed that T-cadherin protein and mRNA expression was downregulated under ER stress conditions. T-cadherin mRNA expression was significantly reduced by activation of IRE1α. Following our previous findings, adiponectin increased EV production from F2T cells, which stably overexpress T-cadherin. Although adiponectin/T-cadherin-mediated and general EV production were downregulated by pharmacological and genetic IRE1α activation, adiponectin/T-cadherin-dependent EV production was more sensitive to ER stress. Pharmacological induction of ER stress in mice almost completely diminished the levels of circulating small EVs. IRE1α activation upregulated the canonical IFN-related pathway, which in turn decreased EV production, suggesting that ER stress–induced IFN-related pathway activation may mediate the general cellular mechanism to arrest exosome production.

Multiple clinical studies have demonstrated that adiponectin, especially its multimeric forms, is a pleiotropic circulating factor, and the concentration of adiponectin declines with increasing weight (31, 32, 33, 34, 35). We have previously demonstrated that the native adiponectin in human or murine serum specifically binds to cells expressing T-cadherin but not to cells expressing other receptor candidates, such as AdipoR1, AdipoR2, or calreticulin (13, 36, 37). Organ protection by adiponectin has been reported to require the presence of T-cadherin in several different mouse models, including heart failure, atherosclerosis, revascularization, muscle regeneration, and renal ischemia‒reperfusion models (16, 17, 38, 39, 40). The present study suggested that T-cadherin is susceptible to ER stress at both the mRNA and protein levels. Interestingly, it has been reported that ER stress in adipocytes attenuates the folding and multimer assembly of adiponectin, which largely represents an overnutrition-induced decrease in multimer adiponectin in plasma (41). Therefore, these findings suggest that adiponectin and T-cadherin are coordinately regulated under ER stress conditions in a localization-dependent manner.

The present study demonstrated that ER stress reduced EV production in multiple types of cells and in mice. TM injection in mice caused almost complete diminishment of circulating EVs. Because half of the circulating EVs depend on the adiponectin/T-cadherin system (14), the adiponectin/T-cadherin-dependent EV production may have been affected by TM injection in mice. ER stress was evident in the liver but not in tissues where T-cadherin is abundantly expressed, such as the heart, aorta, and muscle. Therefore, both adiponectin/T-cadherin-dependent and adiponectin/T-cadherin-independent EV production were likely downregulated.

Although the T-cadherin mRNA sequence possesses an IRE1α recognition site, the responsibility of this site for IRE1α-dependent mRNA degradation was not confirmed by a site-directed mutant or by quantitative PCR primers spanning this site (data not shown). Similarly, RNA-Seq analysis indicated that there was little difference in the expression of genes with or without this recognition site after XBP1 depletion (Table S2). It has been reported that the presence of an IRE1α recognition site is not sufficient for IRE1α-dependent mRNA degradation (42). In the present study, the time-course experiment showed that T-cadherin mRNA decreased later than *XBP1* splicing (Fig. S1, *F* and *G*). Although T-cadherin mRNA is a target of IRE1α-mediated decay, it may not be a superior substrate to the typical substrate, namely, *XBP1* mRNA.

One fundamental role of ER stress response may depend on protein kinase RNA-like ER kinase, which globally represses translation through the phosphorylation of eukaryotic initiation factor 2 alpha (43). The present results suggested that specific inhibition of IRE1 RNase activity did not rescue T-cadherin protein expression, but it significantly rescued T-cadherin mRNA expression. The repression of T-cadherin protein translation by eukaryotic initiation factor 2 alpha may account for this attenuated protein expression.

A previous study on the regulation of T-cadherin protein under ER stress conditions has reported that treatment with chemical inducers of ER stress for less than 9 h upregulates T-cadherin protein in cultured endothelial cells (44). The present study showed IRE1α-dependent downregulation of T-cadherin mRNA and independent downregulation of T-cadherin protein expression. Downregulated T-cadherin has also been observed in the aorta of obese diabetic db/db mice, a pathological model of hyperglycemia-induced vascular ER stress (13, 45).

In the present study, transcriptome analysis suggested that IRE1α activation by *Xbp1* silencing upregulated the classical IFN pathway. Activation of this viral defense pathway by poly(I:C) resulted in the downregulation of EV production. It has been reported that ER stress activates interferon regulatory factor 3 (46). Such IFN-stimulated innate immunity has been reported to downregulate exosome biogenesis through ISGylation of TSG101, a key component of the endosomal sorting complex 1 required for transport (26). In addition, a low dose of TM decreases exosome production in carcinoma cells (47), and Xbp1 depletion significantly decreases the numbers of exosomes and exosome markers, such as TSG101 and CD63, produced from tumor cells (48). Therefore, halting exosome production under ER stress conditions may be a universal cellular response to limit stress within cells.

In summary, the present study suggested that T-cadherin mRNA is downregulated by IRE1α activation and that its protein is downregulated by other pathways under ER stress. In addition, the present study demonstrated that ER stress decreases exosome production through adiponectin/T-cadherin-dependent and adiponectin/T-cadherin-independent pathways.

## Experimental procedures

### Stable cell lines

To generate CHO cells stably overexpressing T-cadherin (CHO-T cells) (13), HEK293T cells stably overexpressing T-cadherin (HEK293TT cells), and F2 cells stably overexpressing T-cadherin (F2T cells) (14), cDNA encoding full-length mouse T-cadherin was subcloned into pMXs-puro, and the resultant vector was used to transfect Plat-E cells and generate recombinant retroviruses. CHO cells, HEK293T cells, and F2 cells were infected with the recombinant retroviruses and selected in growth medium containing 2 μg/ml puromycin.

### EV isolation

EVs were isolated from the cell culture supernatant as described previously (14, 49). Briefly, cells were cultured, and the conditioned medium was collected and centrifuged at 800*g* for 10 min to deplete floating cells. The resultant supernatant was ultracentrifuged at an average of 110,000*g* for 2 h, and the exosome pellet was washed with Dulbecco’s PBS at an average of 110,000*g* for 2 h (TLA120.1 rotor; Beckman Coulter). After drying with a vacuum concentrator, the EV pellets were solubilized directly in Laemmli sample buffer and evaluated by Western blot analysis. Plasma EVs were purified by a phosphatidylserine affinity magnetic resin. The heparinized plasma was incubated with thrombin (500 U/ml) for 10 min at room temperature to remove fibrin and then centrifuged at 12,000*g* for 20 min. The EVs were purified from the defibrinated plasma using the MagCapture Exosome Isolation Kit PS (Fujifilm Wako Pure Chemical). The concentration and size distribution of EVs were analyzed by NPA (Nano Sight NS300 System; Quantum Design).

### Animal procedures

Male C57BL6/J mice (9 weeks old) were purchased from CLEA Japan. Mice were housed in cages in a room set at 22 °C under a 12 h light–dark cycle (lights off from 8:00 AM to 8:00 PM). TM was suspended in 0.5% w/v methylcellulose at a concentration of 100 ng/μl. Mice were randomly allocated to the TM group and vehicle group. TM was injected intraperitoneally (1 mg/kg/day; Cayman) once a day for 2 days as previously reported (50, 51, 52, 53). Mice were euthanized 4 h after the second injection, and the aortas, hearts, muscles, and livers were dissected and processed for further analysis. This study was approved by the Ethics Review Committee for Animal Experimentation of Osaka University School of Medicine and conducted under the Guide for the Care and Use of Laboratory Animals published by the United States National Institute of Health.

### Cell culture

CHO-T cells were maintained in Ham's F-12 culture medium (Nacalai) supplemented with 10% fetal bovine serum, 100 U/ml penicillin, and 100 μg/ml streptomycin. F2, F2T, and HEK293TT cells were maintained in Dulbecco's modified Eagle's medium (Nacalai) supplemented with 10% fetal bovine serum, 100 U/ml penicillin, and 100 μg/ml streptomycin. Cells were grown as a monolayer at 37 °C in 95% air and 5% CO_2_. For passages, cells were detached with trypsin/EDTA (Life Technologies) at 37 °C, washed in complete culture medium, and replated.

### Induction of ER stress and cell transfection

UV-F2, HEK293TT, and CHO-T cells were seeded in collagen-coated 12-well plates (Iwaki; catalog no.: 4815-010) at 1 × 10^5^, 5 × 10^5^, and 6 × 10^5^ cells/well. At 24 h after seeding, cells were treated with TG (Wako), TM (Wako), MG (Wako), or 4μ8c (Wako) at the indicated concentrations for 24 h. To deplete Xbp1, CHO-T cells were transfected with *Xbp1* siRNA or negative control siRNA and seeded in 6-well plates (Falcon) at 1.2 × 10^6^ cells/well. The sequence of *Xbp1* siRNA was obtained from previous studies (54). The medium was changed 24 h after seeding, and cells were harvested 24 h after the medium change. To overexpress IRE1α, HEK293TT cells were transfected with pcDNA3.1(−) encoding IRE1α cDNA or empty vector and seeded in 12-well plates (Iwaki) at 5 × 10^5^ cells/well. At 24 h after seeding, the medium was refreshed, and cells were harvested 24 h after the medium change. To assess adiponectin-dependent EV production, F2T cells were seeded in collagen-coated 12-well plates (Iwaki; catalog no.: 4815-010) at 5 × 10^4^ cells/well. At 24 h after seeding, cells were washed two times with PBS. The medium was replaced with medium containing 5% exosome-free adiponectin-knockout mouse serum, and cells were treated with 100 nmol/l TG and 20 μg/ml HMW-APN purified previously for 48 h (13, 14).

### XBP1 splicing

PCR primers encompassing the missing sequences in Xbp1s were used for PCR amplification with Mighty Amp polymerase (Takara). The PCR products were separated by electrophoresis on a 4% agarose gel (UltraPure Agarose; Invitrogen) and visualized by ethidium bromide staining.

### Antibodies

The following primary antibodies were used: goat polyclonal antiadiponectin (catalog no.: AF1119; R&D Systems), goat polyclonal anti-T-cadherin (catalog no.: AF3264; R&D Systems), rabbit polyclonal anti-BiP (catalog no.: 3183S; CST), rabbit monoclonal anti-a-tubulin (catalog no.: 11H10; Cell Signaling Technology), rabbit polyclonal antisyntenin (catalog no.: ab19903; Abcam), rabbit monoclonal anti-TSG101 (catalog no.: ab125011; Abcam), and mouse monoclonal anti-ALIX (catalog no.: ab186428; Abcam). The following secondary antibodies were used: horseradish peroxidase–conjugated donkey antigoat immunoglobulin (R&D Systems) and donkey anti-rabbit immunoglobulin antibody (GE Healthcare).

### Western blot analysis

Whole-cell lysates were loaded onto 4 to 20% gradient SDS-PAGE gels (Bio-Rad) and transferred onto nitrocellulose membranes. The membranes were blocked with Block-One blocking reagent (Nakarai Tesque) and then incubated with primary antibodies using Can Get Signal Solution 1 (TOYOBO) overnight at 4 °C. The membranes were then incubated with secondary antibodies conjugated with horseradish peroxidase using Can Get Signal Solution 2 (TOYOBO) for 60 min at room temperature. Chemiluminescence signals developed with Chemi-Lumi One Super (Nakarai Tesque) were visualized by a ChemiDoc Touch and quantitated using ImageLab software (Bio-Rad).

### Quantitative real-time PCR

Total RNA was isolated from mouse tissues using RNA STAT-60 (Tel-Test) according to the manufacturer’s protocol. First-strand cDNA was synthesized using ReverTra Ace qPCR RT Master Mix (TOYOBO). Quantitative real-time PCR amplification was conducted with the Real-time PCR ViiA7 (Applied Biosystems) using Power SYBR Green PCR Master Mix (Applied Biosystems) according to the manufacturer’s protocol. The primer sequences are listed in Table S3.

### *Xbp1* silencing

The *Xbp1* gene was silenced by transfecting the following sequence-specific siRNA: Forward_ r(GAG ACG GAG UCC AAG GGA A)dTdT and Reverse_ r(UUC CCU UGG ACU CCG UCU C)dTdG.

### RNA-Seq

The qualities of the total RNA extracts were analyzed using an RNA 6000 Pico Kit (Agilent Technologies), and the samples for RNA-Seq analysis had RNA integrity numbers of 8.0 or more. RNA samples were sequenced on a HiSeq 2500 (Illumina), and raw count and fragments per kilobase of exon per million mapped reads data were obtained. The data were loaded into the iDEP version 0.96 web application. Gene expression analysis was conducted using the DESeq2 package in R. Differentially expressed genes between *Xbp1* siRNA-transfected CHO-T cells and negative control siRNA-transfected CHO-T cells were determined based on a false discovery rate <0.05 and |log2-fold change| ≥1. Enriched pathway analyses were performed using Gene Ontology biological process and ingenuity pathway analysis canonical pathway (QIAGEN).

### Quantification and statistical analysis

Statistical analyses were performed using JMP Pro 15 (SAS Institute). *p* Values less than 0.05 were considered statistically significant. The results are expressed as the mean ± SEM from at least three independent biological experiments unless otherwise specified. The methods of statistical tests and sample sizes are provided in the figure legends.

## Data availability

We submitted all raw datasets, except RNA-Seq, to DRYAD (DOI: https://doi.org/10.5061/dryad.fttdz08xc). RNA-Seq datasets were deposited to the National Center for Biotechnology Information under accession number GSE221308 (reviewers token: etkpwckwbxchbeh).

## Supporting information

This article contains supporting information.

## Conflict of interest

K. F. is an employee of Kowa Company Ltd. S. K. belongs to the endowed department by Takeda Pharmaceutical Company, Rohto Pharmaceutical Co, Ltd, Sanwa Kagaku Kenkyusho Co, Ltd, FUJI OIL HOLDINGS INC, and Kobayashi Pharmaceutical Co, Ltd. N. M. belongs to the endowed department by Kowa Company Ltd. All other authors declare that they have no conflicts of interest with the contents of this article.
