# Supplementary material for: ER stress decreases exosome production through adiponectin/T-cadherin-dependent and -independent pathways
Source: J Biol Chem. 2023 Jul 29;299(9):105114. doi: 10.1016/j.jbc.2023.105114 (PMC10474463; doi:10.1016/j.jbc.2023.105114)
Supplement: Supplemental Figures [file mmc2.pptx]

## Slide 1
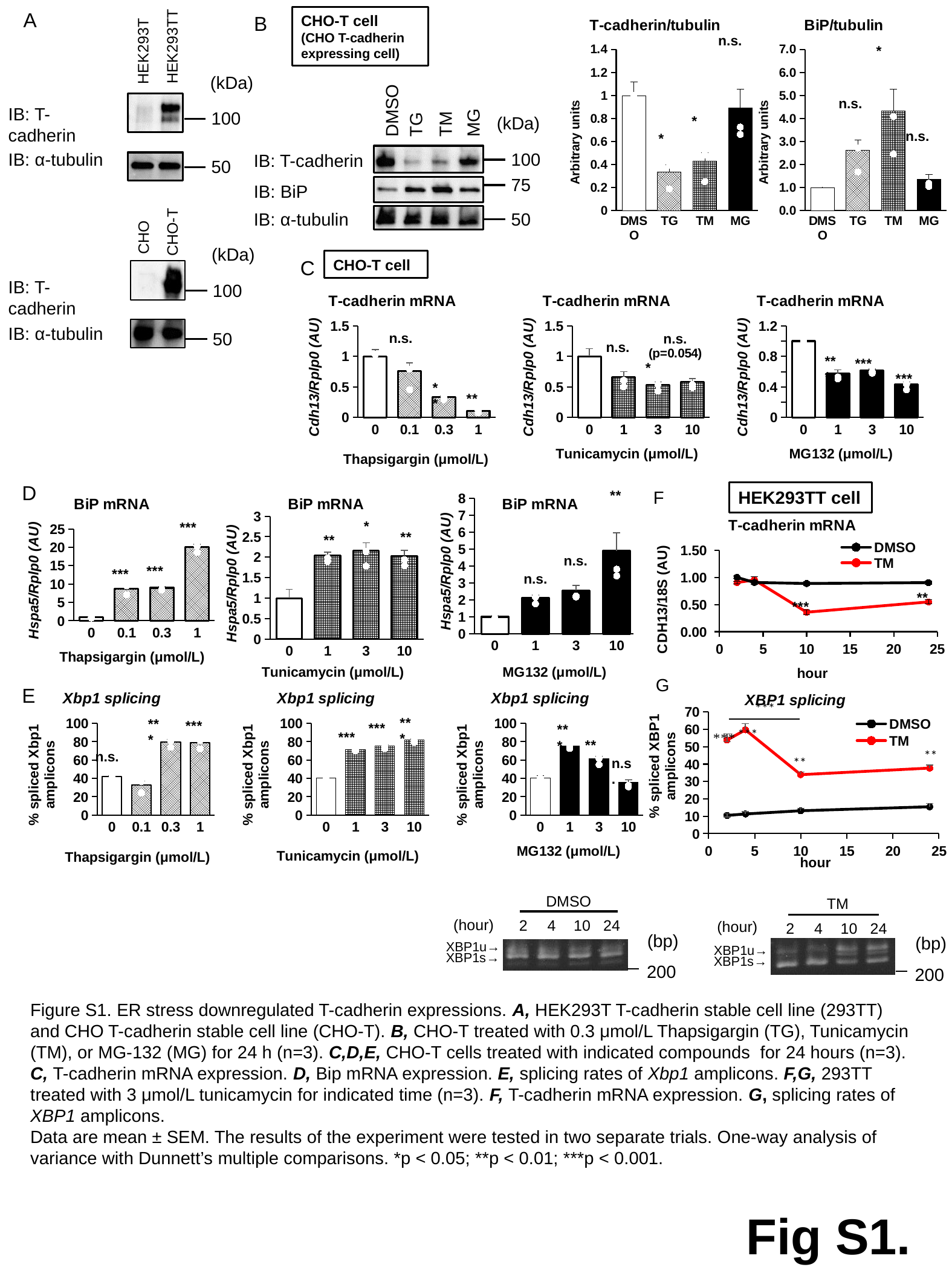

A
### Chart: T-cadherin/tubulin
| Category | | | | |
|---|---|---|---|---|
| DMSO | 1.0 | 0.7440377266797714 | 1.0032657983957238 | 1.2526964749245046 |
| TG | 0.3369593837654954 | 0.18861237958307558 | 0.39238113489335863 | 0.42988463682005196 |
| TM | 0.42955200416523615 | 0.2524867064765838 | 0.48587051949292315 | 0.5502987865262017 |
| MG | 0.8942269290097178 | 0.6628399162166162 | 1.2936548085309976 | 0.7261860622815396 |
### Chart: BiP/tubulin
| Category | | | | |
|---|---|---|---|---|
| DMSO | 1.0 | 0.4974608909153091 | 1.3152995394169817 | 1.187239569667709 |
| TG | 2.6293329881257126 | 1.6802670945824143 | 3.490477374992583 | 2.7172544948021393 |
| TM | 4.337944396990595 | 2.456259762218523 | 6.457395259160831 | 4.100178169592432 |
| MG | 1.3681032735857246 | 1.187483611946275 | 1.866408244951642 | 1.0504179638592572 |B
CHO-T cell
(CHO T-cadherin
expressing cell)
HEK293TT
HEK293T
(kDa)
DMSO
TG
TM
MG
IB: T-cadherin
100
(kDa)
IB: α-tubulin
100
IB: T-cadherin
50
75
IB: BiP
CHO
CHO-T
50
IB: α-tubulin
(kDa)
C
CHO-T cell
IB: T-cadherin
100
### Chart: T-cadherin mRNA
| Category | | | | |
|---|---|---|---|---|
| 0 | 1.0 | 0.7499912605670486 | 1.2183643972836369 | 1.0316443421493142 |
| 0.1 | 0.7599054702642682 | 1.0316443421493142 | 0.7927542926827402 | 0.4553177759607506 |
| 0.3 | 0.3347970473264705 | 0.2921833126443858 | 0.3474664742817681 | 0.36474135505325744 |
| 1 | 0.1110397267140817 | 0.13725643458505526 | 0.11702962524688412 | 0.07883312031030569 |
### Chart: T-cadherin mRNA
| Category | | | | |
|---|---|---|---|---|
| 0 | 1.0 | 1.3033040074264872 | 0.8718631718995293 | 0.8248328206739837 |
| 1 | 0.6602099620122651 | 0.8718631718995293 | 0.6080128184655231 | 0.500753895671743 |
| 3 | 0.53595210752442 | 0.5366947366426421 | 0.4329203865306761 | 0.638241199399942 |
| 10 | 0.5818490581575012 | 0.48706075406109794 | 0.7180586766628677 | 0.5404277437485382 |
### Chart: T-cadherin mRNA
| Category | | | | |
|---|---|---|---|---|
| 0 | 1.0 | 0.9769667679166723 | 0.9974949164558912 | 1.0255383156274367 |
| 1 | 0.5757938209971385 | 0.5308519231122266 | 0.690819826587783 | 0.5057097132914057 |
| 3 | 0.6165298240595632 | 0.5809079157638597 | 0.6013935891721272 | 0.6672879672427029 |
| 10 | 0.43400131506238443 | 0.437204875374447 | 0.36258254867339595 | 0.5022165211393105 |IB: α-tubulin
50
D
HEK293TT cell
F
### Chart: T-cadherin mRNA
| Category | | |
|---|---|---|G
### Chart: XBP1 splicing
| Category | | |
|---|---|---|
### Chart: BiP mRNA
| Category | | | | |
|---|---|---|---|---|
| 0 | 1.0 | 0.8044860314055776 | 1.4500868159364537 | 0.7454271526579687 |
| 0.1 | 8.689400686242461 | 9.488231934735394 | 9.488231934735394 | 7.091738189256599 |
| 0.3 | 8.939782517635946 | 8.610752230864344 | 8.317438399162555 | 9.891156922880938 |
| 1 | 20.140844798627832 | 18.585933890184617 | 20.338470388077518 | 21.49813011762137 |
### Chart: BiP mRNA
| Category | | | | |
|---|---|---|---|---|
| 0 | 1.0 | 1.520504903160434 | 0.7602524515802184 | 0.7192426452593474 |
| 1 | 2.0394743219089517 | 1.9786966718873664 | 1.8980927203698634 | 2.241633573469625 |
| 3 | 2.1645758030112847 | 2.574960798350679 | 2.135465319778175 | 1.783301290905 |
| 10 | 2.028920325384981 | 1.9379756832141866 | 1.7957051323001032 | 2.353080160640654 |
### Chart: BiP mRNA
| Category | | | | |
|---|---|---|---|---|
| 0 | 1.0 | 0.883145106625246 | 1.1898027408901612 | 0.9270521524845925 |
| 1 | 2.1184779404536216 | 2.1595375568133597 | 2.4296060512970126 | 1.766290213250492 |
| 3 | 2.561166221179847 | 3.2732468508634045 | 2.2356934842869864 | 2.1745583283891503 |
| 10 | 4.897570264633316 | 3.812460544002709 | 3.412247768806124 | 7.468002481091115 |
### Chart: Xbp1 splicing
| Category | | | | |
|---|---|---|---|---|
| 0 | 42.31792173804396 | 42.901667026121885 | 43.45654506830064 | 40.59555311970933 |
| 0.1 | 33.03611304299128 | 38.54439727573622 | 36.39836725304198 | 24.165574600195644 |
| 0.3 | 79.86637972305861 | 73.76254918320487 | 85.17453419809644 | 80.66205578787452 |
| 1 | 78.96042238055078 | 81.3571668350799 | 83.48015310818178 | 72.04394719839067 |
### Chart: Xbp1 splicing
| Category | | | | |
|---|---|---|---|---|
| 0 | 40.062127581201274 | 38.99034502852625 | 42.05946316629396 | 39.136574548783614 |
| 1 | 71.34135405259964 | 72.14632107037706 | 70.05982362161345 | 71.81791746580839 |
| 3 | 75.36789966448248 | 77.84964363762276 | 72.43172308482913 | 75.82233227099556 |
| 10 | 81.81434705670434 | 84.63255567908327 | 81.53846104754474 | 79.27202444348498 |
### Chart: Xbp1 splicing
| Category | | | | |
|---|---|---|---|---|
| 0 | 40.5739792396867 | 35.440173499357016 | 45.87242275440324 | 40.40934146529984 |
| 1 | 75.45096630181257 | 72.55758603253358 | 77.36587437173496 | 76.42943850116916 |
| 3 | 61.21079696771491 | 54.950599430837684 | 61.85802598077922 | 66.82376549152784 |
| 10 | 35.41286766815016 | 32.26309372186974 | 43.200145296416125 | 30.775363986164628 |E
DMSO
TM
(hour)
2
4
10
24
(hour)
2
4
10
24
(bp)
(bp)
XBP1u→
XBP1u→
XBP1s→
XBP1s→
200
200
Figure S1. ER stress downregulated T-cadherin expressions. A, HEK293T T-cadherin stable cell line (293TT) and CHO T-cadherin stable cell line (CHO-T). B, CHO-T treated with 0.3 μmol/L Thapsigargin (TG), Tunicamycin (TM), or MG-132 (MG) for 24 h (n=3). C,D,E, CHO-T cells treated with indicated compounds for 24 hours (n=3). C, T-cadherin mRNA expression. D, Bip mRNA expression. E, splicing rates of Xbp1 amplicons. F,G, 293TT treated with 3 μmol/L tunicamycin for indicated time (n=3). F, T-cadherin mRNA expression. G, splicing rates of XBP1 amplicons.
Data are mean ± SEM. The results of the experiment were tested in two separate trials. One-way analysis of variance with Dunnett’s multiple comparisons. *p < 0.05; **p < 0.01; ***p < 0.001.
Fig S1.

## Slide 2
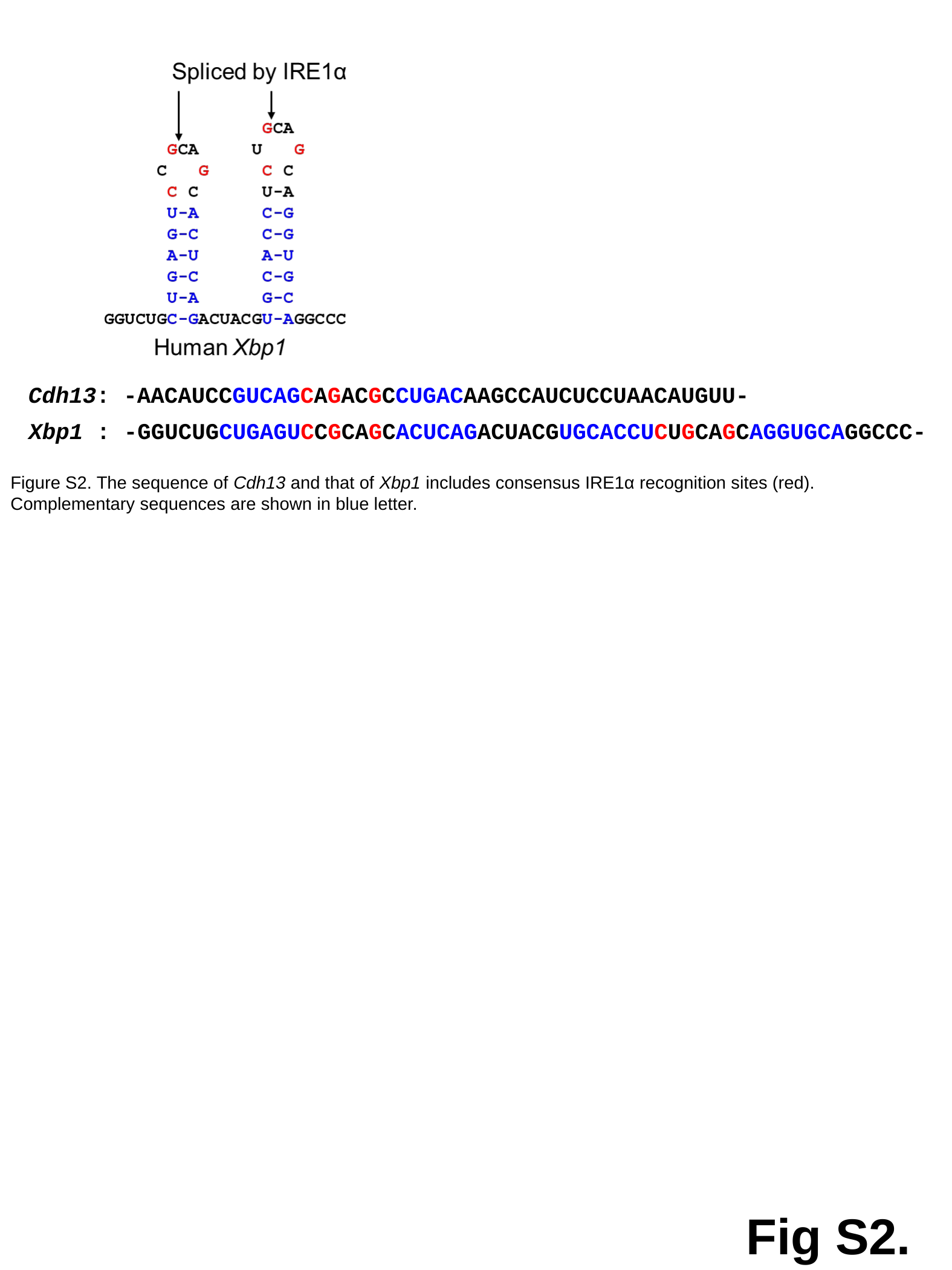

Cdh13: -AACAUCCGUCAGCAGACGCCUGACAAGCCAUCUCCUAACAUGUU-
Xbp1 : -GGUCUGCUGAGUCCGCAGCACUCAGACUACGUGCACCUCUGCAGCAGGUGCAGGCCC-
Figure S2. The sequence of Cdh13 and that of Xbp1 includes consensus IRE1α recognition sites (red). Complementary sequences are shown in blue letter.
Fig S2.

## Slide 3
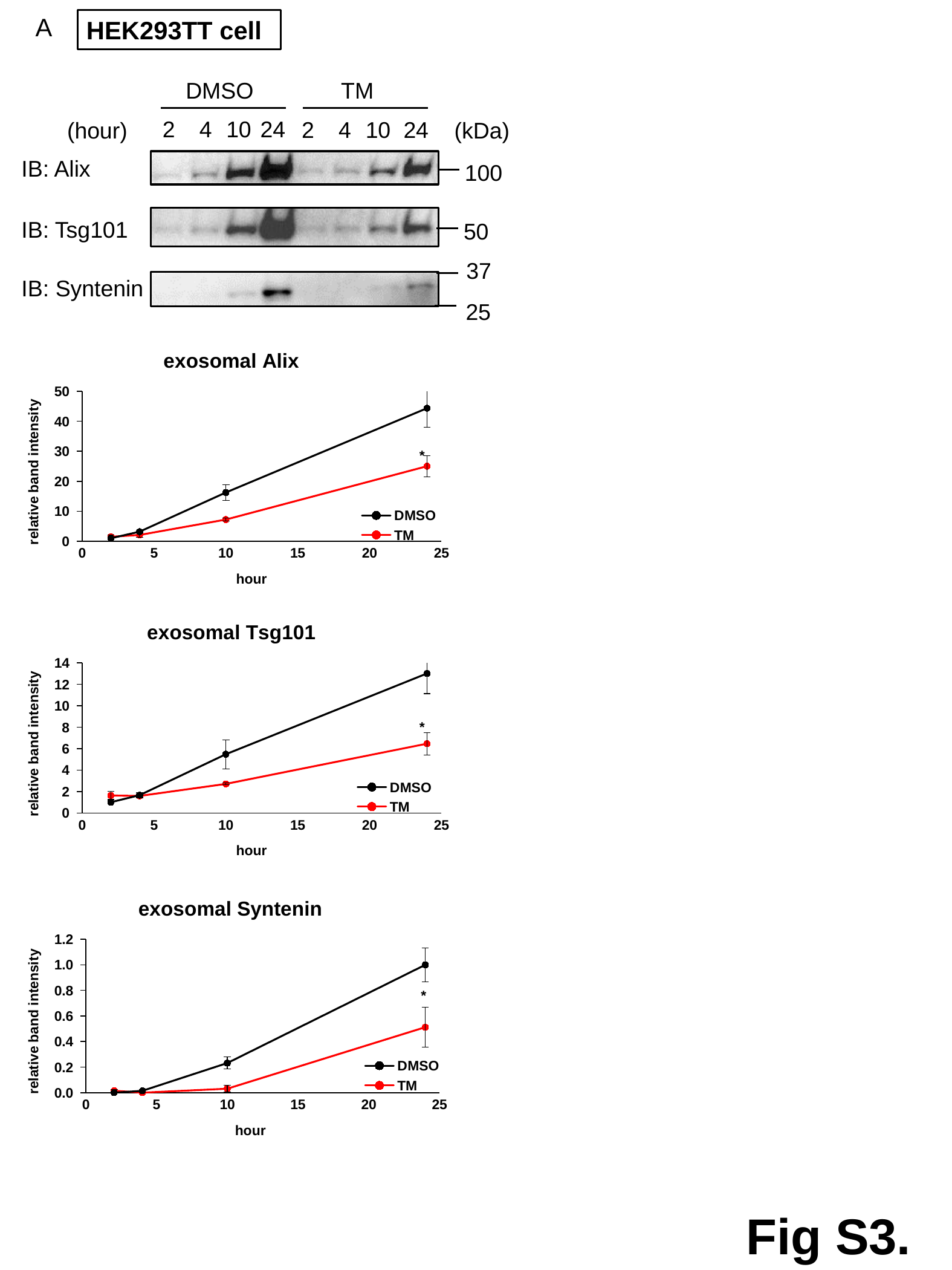

A
HEK293TT cell
DMSO
TM
2
4
10
24
2
4
10
24
(hour)
(kDa)
IB: Alix
100
IB: Tsg101
50
37
IB: Syntenin
25
### Chart: exosomal Alix
| Category | DMSO | TM |
|---|---|---|
### Chart: exosomal Tsg101
| Category | DMSO | TM |
|---|---|---|
### Chart: exosomal Syntenin
| Category | DMSO | TM |
|---|---|---|Fig S3.

## Slide 4
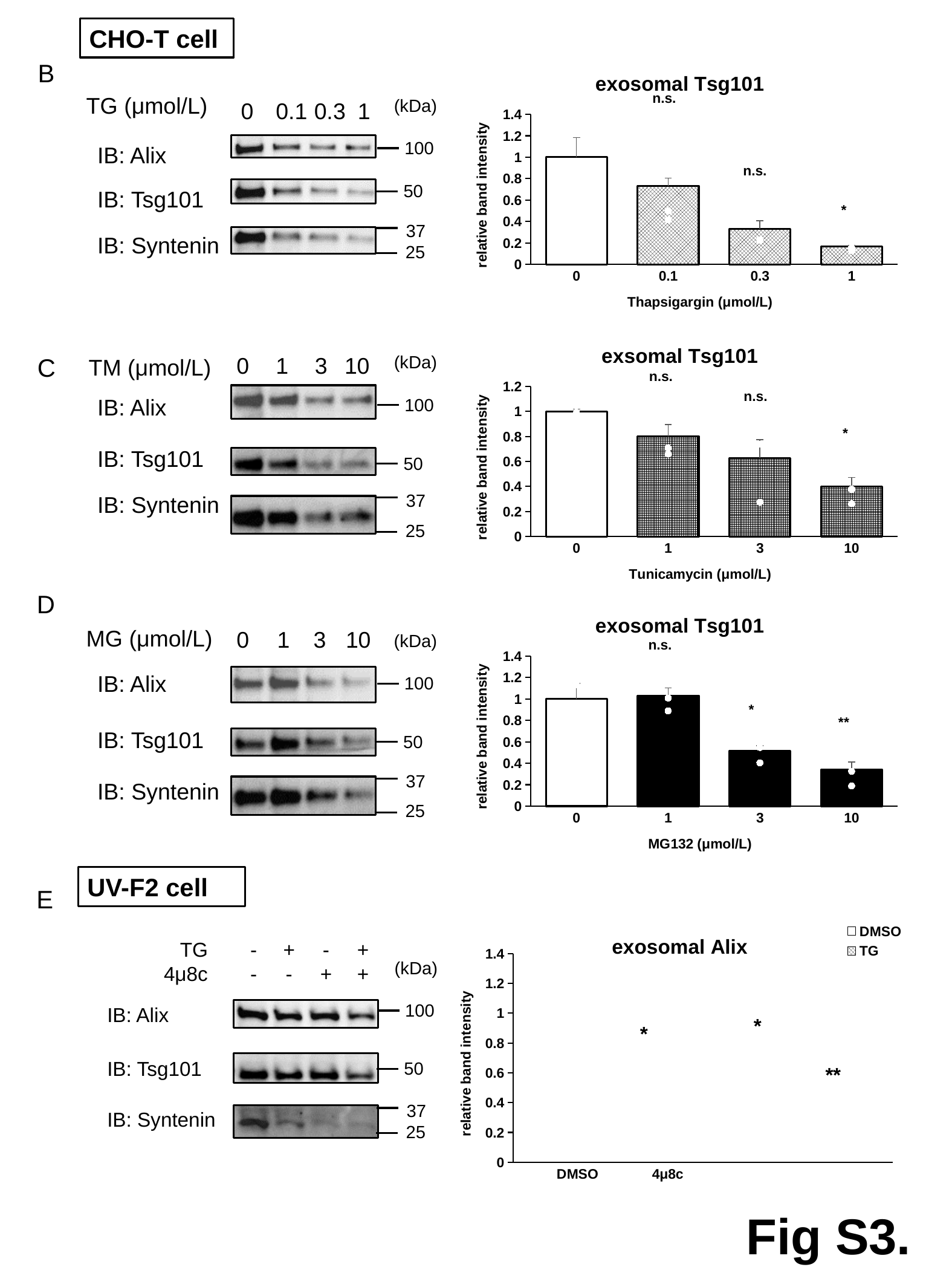

CHO-T cell
### Chart: ALIX
| Category | | | | |
|---|---|---|---|---|
| 0 | 1.0 | 0.4521446271834098 | 1.0098399052377038 | 1.5380154675788864 |
| 0.1 | 0.8955997832410627 | 0.5496876987236742 | 0.3849953326572962 | 1.752116318342218 |
| 0.3 | 0.36525921445032034 | 0.5300616130563682 | 0.23881090468960753 | 0.32690512560498525 |
| 1 | 0.21396111460379766 | 0.06865100622949483 | 0.26266859387196245 | 0.31056374370993567 |B
### Chart: exosomal Tsg101
| Category | | | | |
|---|---|---|---|---|
| 0 | 1.0 | 0.4896730329280518 | 1.2552572482140896 | 1.2550697188578586 |
| 0.1 | 0.7294736169758661 | 0.4991529047771497 | 0.41530449860141655 | 1.2739634475490322 |
| 0.3 | 0.33134353586503806 | 0.536904725739777 | 0.2264197900138573 | 0.23070609184147986 |
| 1 | 0.16593368588073368 | 0.1296826839715563 | 0.156075250097562 | 0.2120431235730827 |TG (μmol/L)
(kDa)
0
0.1
0.3
1
100
IB: Alix
50
IB: Tsg101
37
IB: Syntenin
25
### Chart: ALIX
| Category | | | | |
|---|---|---|---|---|
| 0 | 1.0 | 1.1939712600454822 | 0.7020254995123715 | 1.1040032404421463 |
| 1 | 0.7623489783606011 | 0.8336550714481786 | 0.4140869964984478 | 1.0393048671351768 |
| 3 | 0.49207817529482745 | 0.35209233921091215 | 0.6028081600224956 | 0.5213340266510744 |
| 10 | 0.3953651857786546 | 0.43198976984380266 | 0.25761738233869763 | 0.4964884051534635 |
### Chart: exsomal Tsg101
| Category | | | | |
|---|---|---|---|---|
| 0 | 1.0 | 1.0446385570612398 | 0.9889034285984094 | 0.9664580143403506 |
| 1 | 0.7998880292473002 | 0.6595171767181777 | 0.708330691453124 | 1.0318162195705989 |
| 3 | 0.6278739721961781 | 0.27517385820185764 | 0.869413772402582 | 0.7390342859840947 |
| 10 | 0.4002040430885041 | 0.2621876117178067 | 0.3775734723749266 | 0.560851045172779 |(kDa)
0
1
3
10
C
TM (μmol/L)
IB: Alix
100
IB: Tsg101
50
37
IB: Syntenin
25
### Chart: ALIX
| Category | | | | |
|---|---|---|---|---|
| 0 | 1.0 | 0.8185426348105792 | 0.7538734224709703 | 1.4275839427184502 |
| 1 | 1.120481202537335 | 1.0668520552335465 | 0.9952004815152095 | 1.2993910708632488 |
| 3 | 0.5613466792256673 | 0.3849186465983217 | 0.4298273658538492 | 0.8692940252248308 |
| 10 | 0.25729679237392467 | 0.17400443212863334 | 0.3414966837203303 | 0.2563892612728104 |D
### Chart: exosomal Tsg101
| Category | | | | |
|---|---|---|---|---|
| 0 | 1.0 | 0.6440151788639833 | 1.1344944044290068 | 1.2214904167070098 |
| 1 | 1.03077644198246 | 1.0078962538240517 | 1.1952576309507243 | 0.8891754411726044 |
| 3 | 0.5169854899242532 | 0.5475009570148063 | 0.5992826717160286 | 0.4041728410419246 |
| 10 | 0.3383282987194276 | 0.32519651665846977 | 0.5014661402711189 | 0.18832223922869415 |MG (μmol/L)
0
1
3
10
(kDa)
IB: Alix
100
IB: Tsg101
50
37
IB: Syntenin
25
備忘録
UV-F2 cell
E
### Chart: exosomal Alix
| Category | DMSO | TG | | | |
|---|---|---|---|---|---|
| DMSO | 1.0 | 0.6850620268055585 | 1.2009692216778365 | 0.8944667007923348 | 0.9045640775298287 |
| 4μ8c | 0.699552040216463 | 0.4114121592346715 | 0.730030390290918 | 0.6782858637384471 | 0.6468698263873105 |TG
4μ8c
-
-
+
-
-
+
+
+
(kDa)
100
IB: Alix
Fig3に対応
RNA量は減少していないが、回収手技の関係でサンプル間のブレや、取り損ないがある
IB: Tsg101
50
37
IB: Syntenin
25
Fig S3.

## Slide 5
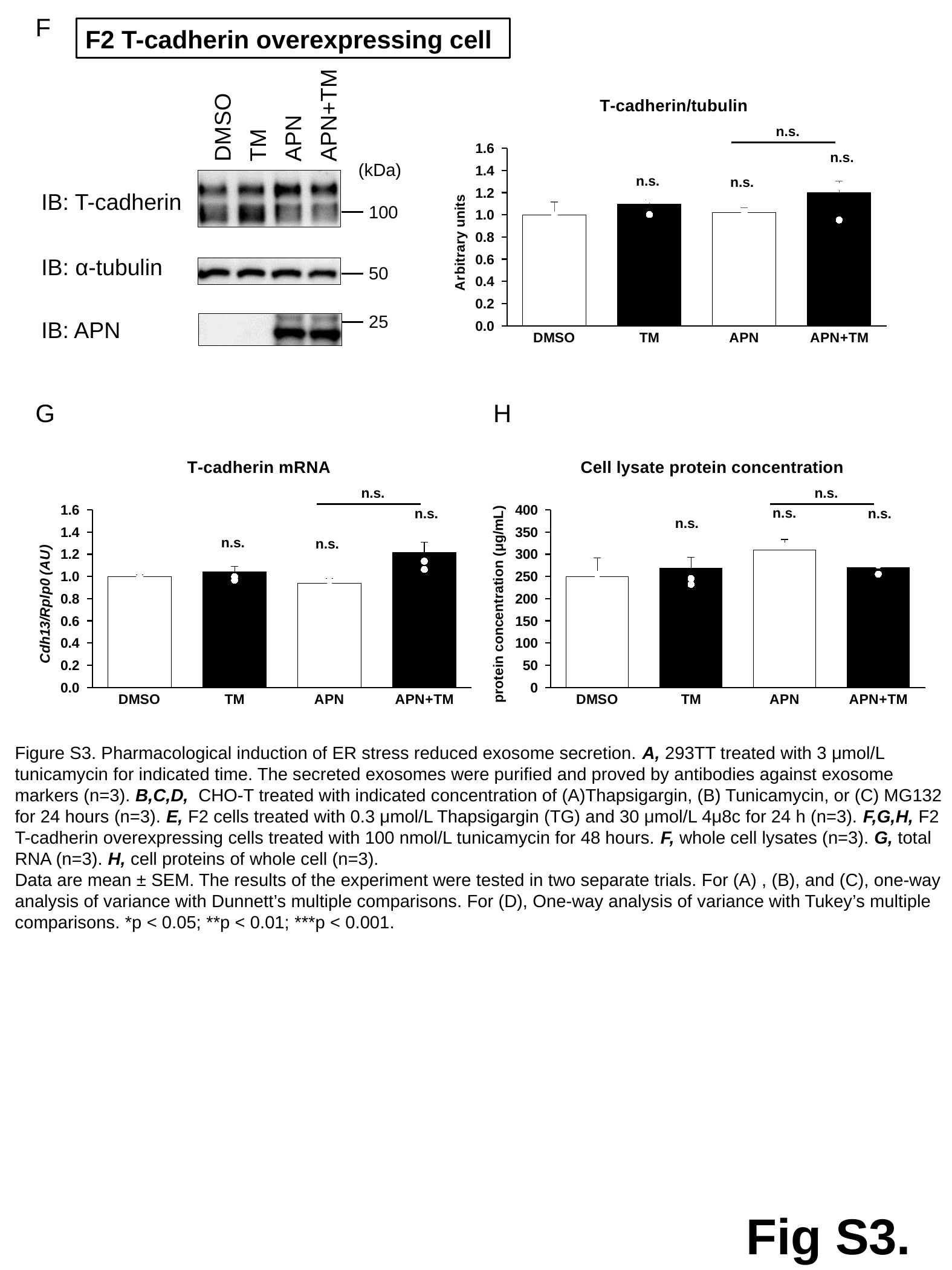

F
F2 T-cadherin overexpressing cell
DMSO
TM
APN
APN+TM
### Chart: T-cadherin/tubulin
| Category | | | | |
|---|---|---|---|---|
| DMSO | 1.0 | 0.756280242122882 | 0.9946547978835649 | 1.2490649599935528 |
| TM | 1.0956281558296561 | 1.0018572372228611 | 1.1464173882342796 | 1.1386098420318271 |
| APN | 1.0204119955047315 | 0.9257545162459775 | 1.0316678457762167 | 1.1038136244920005 |
| APN+TM | 1.1984291449702331 | 0.9523708309991902 | 1.2540193014939875 | 1.388897302417521 |(kDa)
IB: T-cadherin
100
IB: α-tubulin
50
25
IB: APN
G
H
### Chart: T-cadherin mRNA
| Category | | | | |
|---|---|---|---|---|
| DMSO | 1.0 | 1.0388105813661102 | 0.9708589292283535 | 0.9903304894055366 |
| TM | 1.0415008709422018 | 0.9647454723942234 | 1.1617257364557203 | 0.9980314039766616 |
| APN | 0.9378494962565367 | 1.016338690015735 | 0.835829495611612 | 0.9613803031422629 |
| APN+TM | 1.2147796230518773 | 1.1370472953505648 | 1.4446851025178837 | 1.0626064712871837 |
### Chart: Cell lysate protein concentration
| Category | | | | |
|---|---|---|---|---|
| DMSO | 249.79066666666668 | 158.92919999999998 | 255.33100000000005 | 335.1118 |
| TM | 268.62780000000004 | 232.06160000000003 | 328.4634 | 245.35840000000005 |
| APN | 309.62626666666665 | 255.33100000000005 | 355.057 | 318.4908 |
| APN+TM | 269.73586666666665 | 255.33100000000005 | 278.6003999999999 | 275.27619999999996 |Figure S3. Pharmacological induction of ER stress reduced exosome secretion. A, 293TT treated with 3 μmol/L tunicamycin for indicated time. The secreted exosomes were purified and proved by antibodies against exosome markers (n=3). B,C,D, CHO-T treated with indicated concentration of (A)Thapsigargin, (B) Tunicamycin, or (C) MG132 for 24 hours (n=3). E, F2 cells treated with 0.3 μmol/L Thapsigargin (TG) and 30 μmol/L 4μ8c for 24 h (n=3). F,G,H, F2 T-cadherin overexpressing cells treated with 100 nmol/L tunicamycin for 48 hours. F, whole cell lysates (n=3). G, total RNA (n=3). H, cell proteins of whole cell (n=3).
Data are mean ± SEM. The results of the experiment were tested in two separate trials. For (A) , (B), and (C), one-way analysis of variance with Dunnett’s multiple comparisons. For (D), One-way analysis of variance with Tukey’s multiple comparisons. *p < 0.05; **p < 0.01; ***p < 0.001.
Fig S3.

## Slide 6
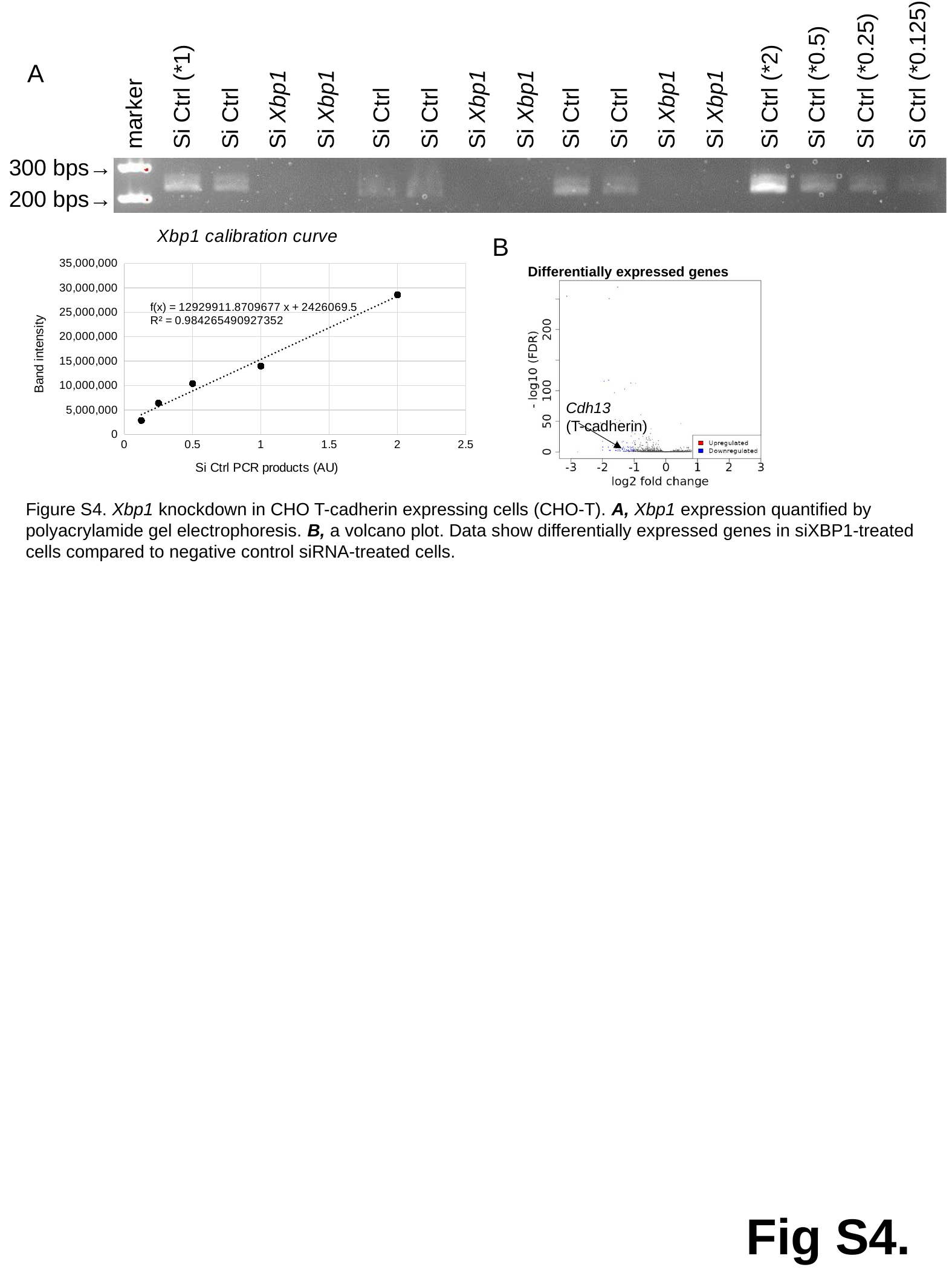

Si Ctrl (*0.5)
Si Ctrl (*0.25)
Si Ctrl (*0.125)
A
Si Ctrl (*2)
Si Xbp1
Si Xbp1
marker
Si Ctrl (*1)
Si Xbp1
Si Xbp1
Si Ctrl
Si Ctrl
Si Ctrl
Si Xbp1
Si Xbp1
Si Ctrl
Si Ctrl
300 bps→
200 bps→
### Chart: Xbp1 calibration curve
| Category | |
|---|---|B
Differentially expressed genes
Cdh13
(T-cadherin)
Figure S4. Xbp1 knockdown in CHO T-cadherin expressing cells (CHO-T). A, Xbp1 expression quantified by polyacrylamide gel electrophoresis. B, a volcano plot. Data show differentially expressed genes in siXBP1-treated cells compared to negative control siRNA-treated cells.
Fig S4.

## Slide 7
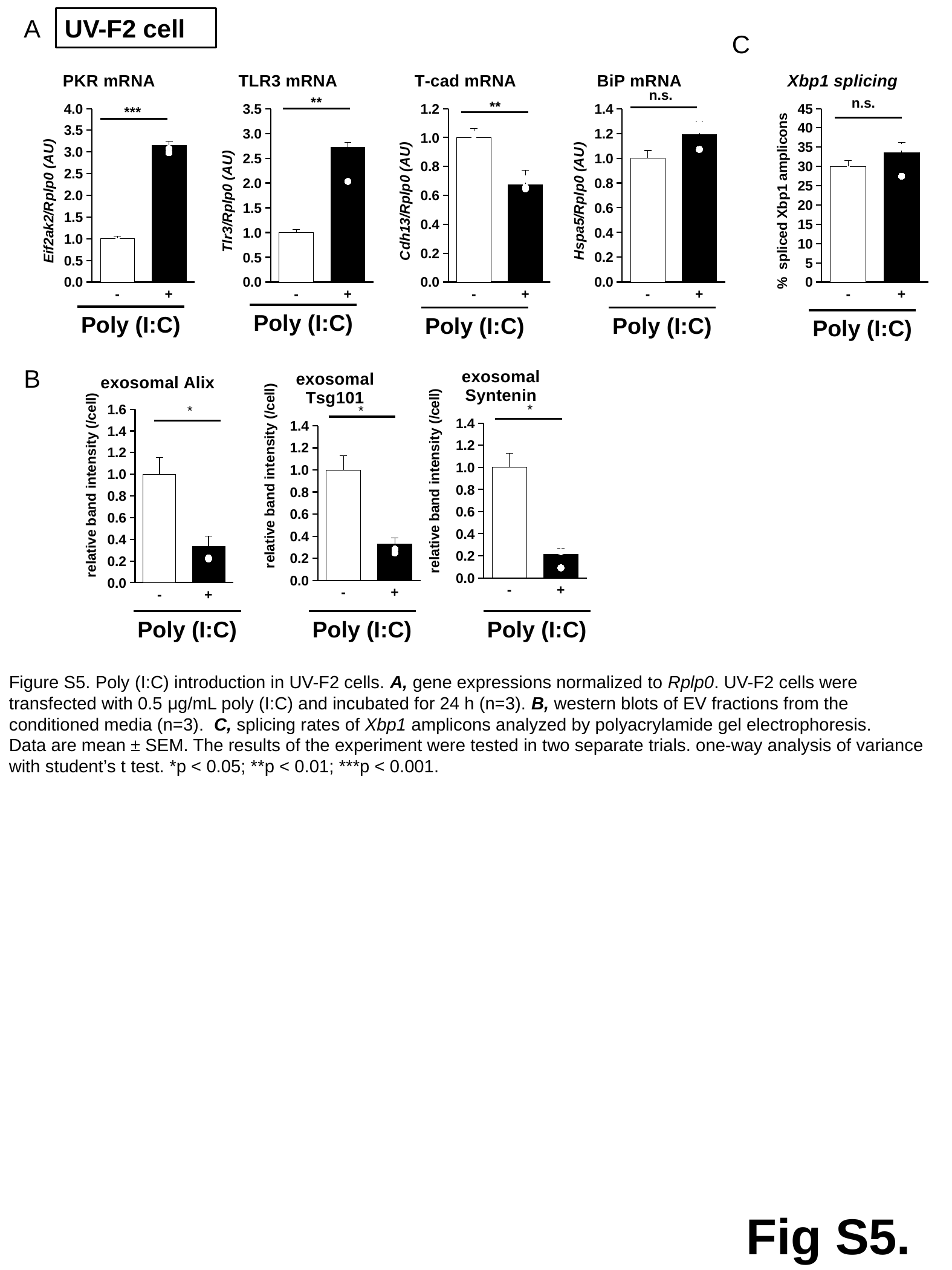

A
UV-F2 cell
C
### Chart: PKR mRNA
| Category | | | | |
|---|---|---|---|---|
| - | 1.0 | 0.9371031423244124 | 0.913486030701912 | 1.1494108269736758 |
| + | 3.1508084015172697 | 3.0772188311507853 | 2.9802688918553266 | 3.3949374815456976 |
### Chart: TLR3 mRNA
| Category | | | | |
|---|---|---|---|---|
| - | 1.0 | 0.9144095781296231 | 1.1902860380704794 | 0.8953043837998977 |
| + | 2.71717105986938 | 3.2323104879972697 | 2.888745405539901 | 2.03045728607097 |
### Chart: T-cad mRNA
| Category | | | | |
|---|---|---|---|---|
| - | 1.0 | 1.1011733910998025 | 0.8803123886629522 | 1.0185142202372455 |
| + | 0.6713806942105685 | 0.655028288067691 | 0.6454526462523955 | 0.7136611483116188 |
### Chart: BiP mRNA
| Category | | | | |
|---|---|---|---|---|
| - | 1.0 | 1.1795637120370726 | 0.93877689261256 | 0.8816593953503675 |
| + | 1.191640799391663 | 1.2731149393635897 | 1.2311704072302165 | 1.070637051581183 |
### Chart: Xbp1 splicing
| Category | | | | |
|---|---|---|---|---|
| - | 29.975096310164172 | 29.124265662977773 | 27.100722127781356 | 33.70030113973338 |
| + | 33.5808014452761 | 27.491280003161155 | 34.93329864251428 | 38.317825690152866 |Poly (I:C)
Poly (I:C)
Poly (I:C)
Poly (I:C)
Poly (I:C)
### Chart: exosomal Syntenin
| Category | | | | |
|---|---|---|---|---|
| - | 1.0 | 1.2881362043484479 | 1.1892386497945702 | 0.522625145856982 |
| + | 0.21352431640651445 | 0.2374954363530754 | 0.3127766681614808 | 0.09030084470498707 |
### Chart: exosomal Tsg101
| Category | | | | |
|---|---|---|---|---|
| - | 1.0 | 1.3041559224037613 | 0.7988371350712966 | 0.8970069425249421 |
| + | 0.3308019093238912 | 0.2482573152574083 | 0.46104929425007257 | 0.2830991184641926 |B
### Chart: exosomal Alix
| Category | | | | |
|---|---|---|---|---|
| - | 1.0 | 1.3528150752252315 | 0.9517765294084177 | 0.695408395366351 |
| + | 0.3358080272376183 | 0.21955380440046451 | 0.5592113551128562 | 0.2286589221995342 |Poly (I:C)
Poly (I:C)
Poly (I:C)
Figure S5. Poly (I:C) introduction in UV-F2 cells. A, gene expressions normalized to Rplp0. UV-F2 cells were transfected with 0.5 μg/mL poly (I:C) and incubated for 24 h (n=3). B, western blots of EV fractions from the conditioned media (n=3). C, splicing rates of Xbp1 amplicons analyzed by polyacrylamide gel electrophoresis.
Data are mean ± SEM. The results of the experiment were tested in two separate trials. one-way analysis of variance with student’s t test. *p < 0.05; **p < 0.01; ***p < 0.001.
Fig S5.

## Slide 8
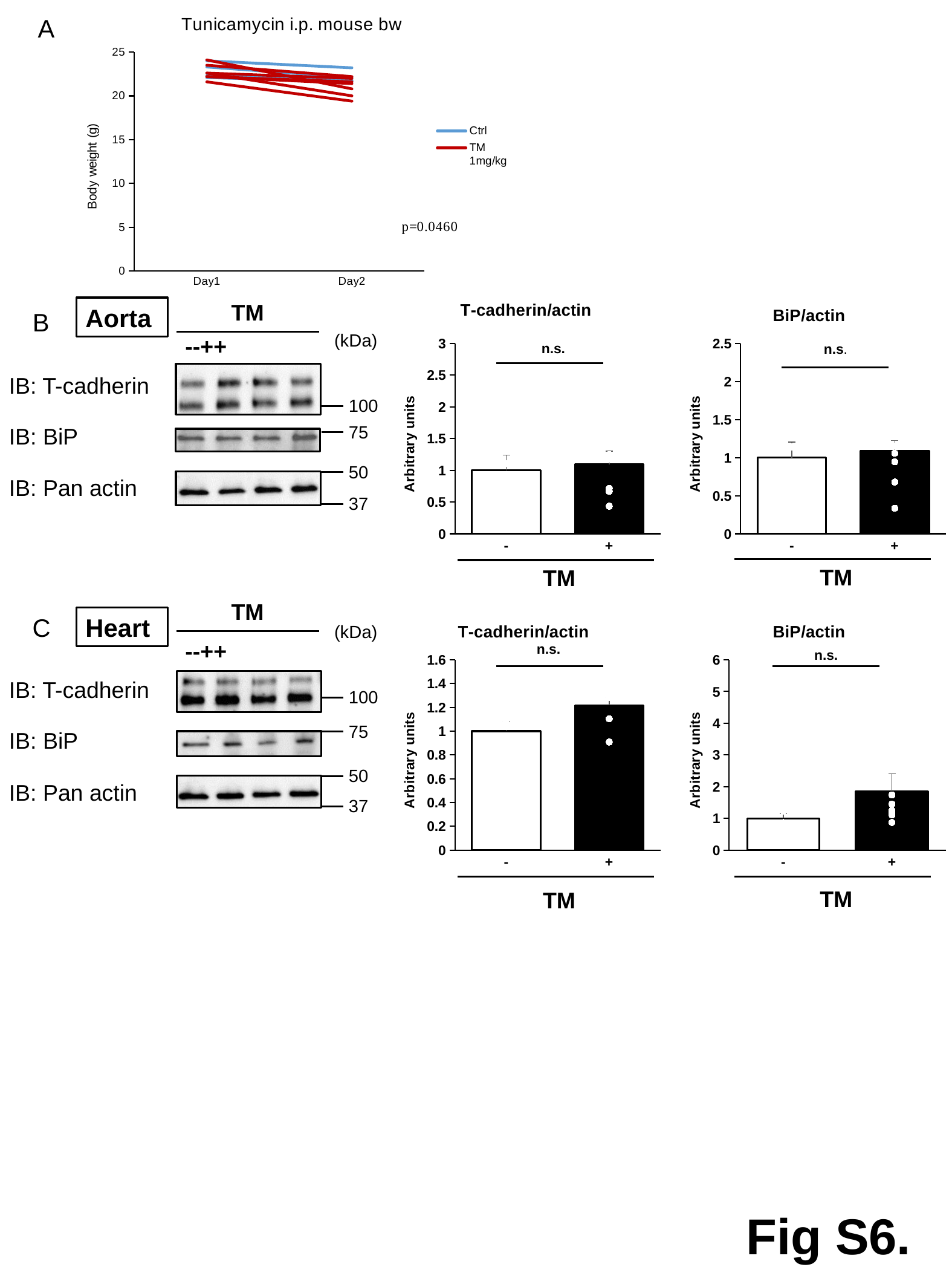

### Chart: Tunicamycin i.p. mouse bw
| Category | Ctrl | | | | | TM
1mg/kg | | | | | | |
|---|---|---|---|---|---|---|---|---|---|---|---|---|
| Day1 | 22.3 | 22.1 | 23.3 | 22.1 | 24.0 | 22.6 | 21.6 | 24.1 | 22.2 | 22.6 | 23.5 | 22.2 |
| Day2 | 21.8 | 21.6 | 22.1 | 21.7 | 23.2 | 20.0 | 19.4 | 20.8 | 21.4 | 22.0 | 22.2 | 21.6 |A
### Chart: T-cadherin/actin
| Category | | | | | | | | |
|---|---|---|---|---|---|---|---|---|
| - | 1.0 | 0.6125413473164423 | 2.3304154406097037 | 0.7602928119790523 | 1.1052683165839388 | 1.5383879116002461 | 0.8317523154900635 | None |
| + | 1.0958864896246012 | 0.7138459859775379 | 1.2489838239314404 | 2.1225938566288685 | 0.6666958470192612 | 1.5216915928873884 | 1.171841545146243 | 0.4330027222482973 |
### Chart: BiP/actin
| Category | | | | | | | | |
|---|---|---|---|---|---|---|---|---|
| - | 1.0 | 1.1406849223992563 | 1.9137972956920095 | 0.6355691255427557 | 1.2808907671511702 | 1.6860711806657598 | 0.5680102524242028 | None |
| + | 1.090492162541027 | 1.0566067726364528 | 0.9442254436479843 | 1.1498282251638703 | 1.5354167920784492 | 0.6787050316147589 | 1.1517214139742715 | 0.33479055902427984 |TM
Aorta
B
(kDa)
--++
IB: T-cadherin
100
75
IB: BiP
50
IB: Pan actin
37
TM
TM
TM
C
Heart
### Chart: T-cadherin/actin
| Category | | | | | | | |
|---|---|---|---|---|---|---|---|
| - | 1.0 | 1.0753724267071798 | 0.7932367730311148 | 1.0929579126194657 | 1.300479862959672 | 0.7001187674178243 | 1.0378342572647437 |
| + | 1.2173609723070657 | 1.281347716986766 | 1.3037339120162494 | 1.3668720073919294 | 1.1040329639401414 | 1.4125121368285416 | 0.9087076639593095 |
### Chart: BiP/actin
| Category | | | | | | | | |
|---|---|---|---|---|---|---|---|---|
| - | 1.0 | 0.866629073722658 | 1.4042116956696733 | 0.40491010907113867 | 1.2265563742592884 | 1.348305188590353 | 0.7493875586868887 | None |
| + | 1.8523094880155748 | 1.4487473052549291 | 1.2465609031677187 | 1.1100910537945552 | 1.737125001155058 | 0.8701830926272651 | 1.161166572365653 | 5.392292487743844 |(kDa)
--++
IB: T-cadherin
100
75
IB: BiP
50
IB: Pan actin
37
TM
TM
Fig S6.

## Slide 9
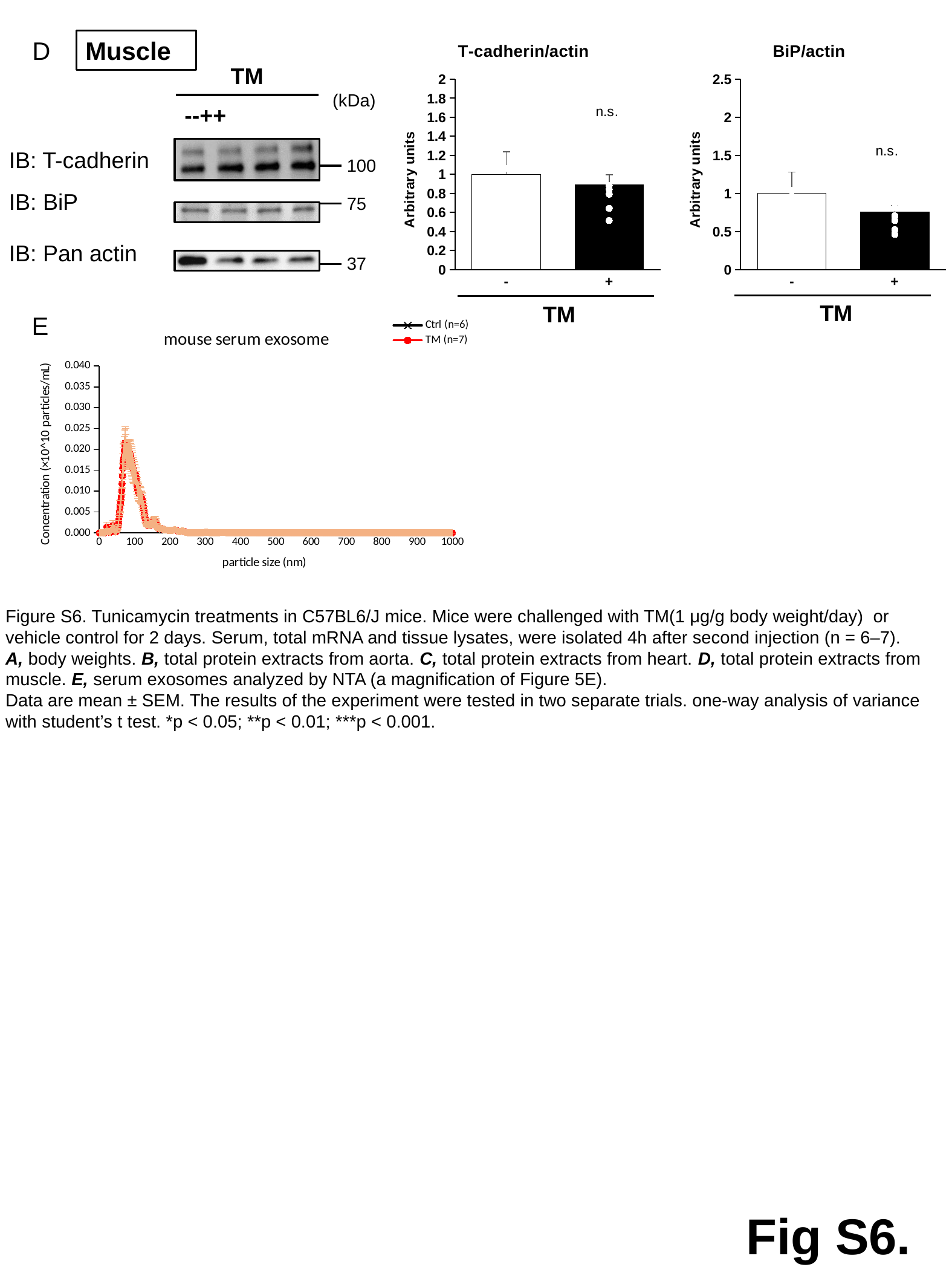

### Chart: T-cadherin/actin
| Category | | | | | | | | |
|---|---|---|---|---|---|---|---|---|
| - | 1.0 | 1.6919026089609135 | 1.7480685561364486 | 0.27166011463252143 | 0.8783593301581156 | 0.34824201924954623 | 1.0617673708624542 | None |
| + | 0.8899440191039625 | 0.8820732843285893 | 0.7918445233506177 | 1.1896497920510412 | 0.8436182821254998 | 0.5138489095782677 | 1.3660679582355941 | 0.642505384058127 |
### Chart: BiP/actin
| Category | | | | | | | | |
|---|---|---|---|---|---|---|---|---|
| - | 1.0 | 1.3950479460094898 | 2.2735677704864266 | 0.3413082116687684 | 0.6870343960160417 | 0.2648051450954762 | 1.0382365307237973 | None |
| + | 0.7560401102603059 | 1.1567383342446216 | 0.6442782294847118 | 0.9888246158802857 | 0.4614457256113325 | 0.7051163597480049 | 0.8132225077180606 | 0.5226549991351245 |D
Muscle
TM
(kDa)
--++
IB: T-cadherin
100
IB: BiP
75
IB: Pan actin
37
TM
TM
E
### Chart: mouse serum exosome
| Category | Ctrl (n=6) | TM (n=7) |
|---|---|---|Figure S6. Tunicamycin treatments in C57BL6/J mice. Mice were challenged with TM(1 μg/g body weight/day) or vehicle control for 2 days. Serum, total mRNA and tissue lysates, were isolated 4h after second injection (n = 6–7).
A, body weights. B, total protein extracts from aorta. C, total protein extracts from heart. D, total protein extracts from muscle. E, serum exosomes analyzed by NTA (a magnification of Figure 5E).
Data are mean ± SEM. The results of the experiment were tested in two separate trials. one-way analysis of variance with student’s t test. *p < 0.05; **p < 0.01; ***p < 0.001.
Fig S6.

## Slide 10
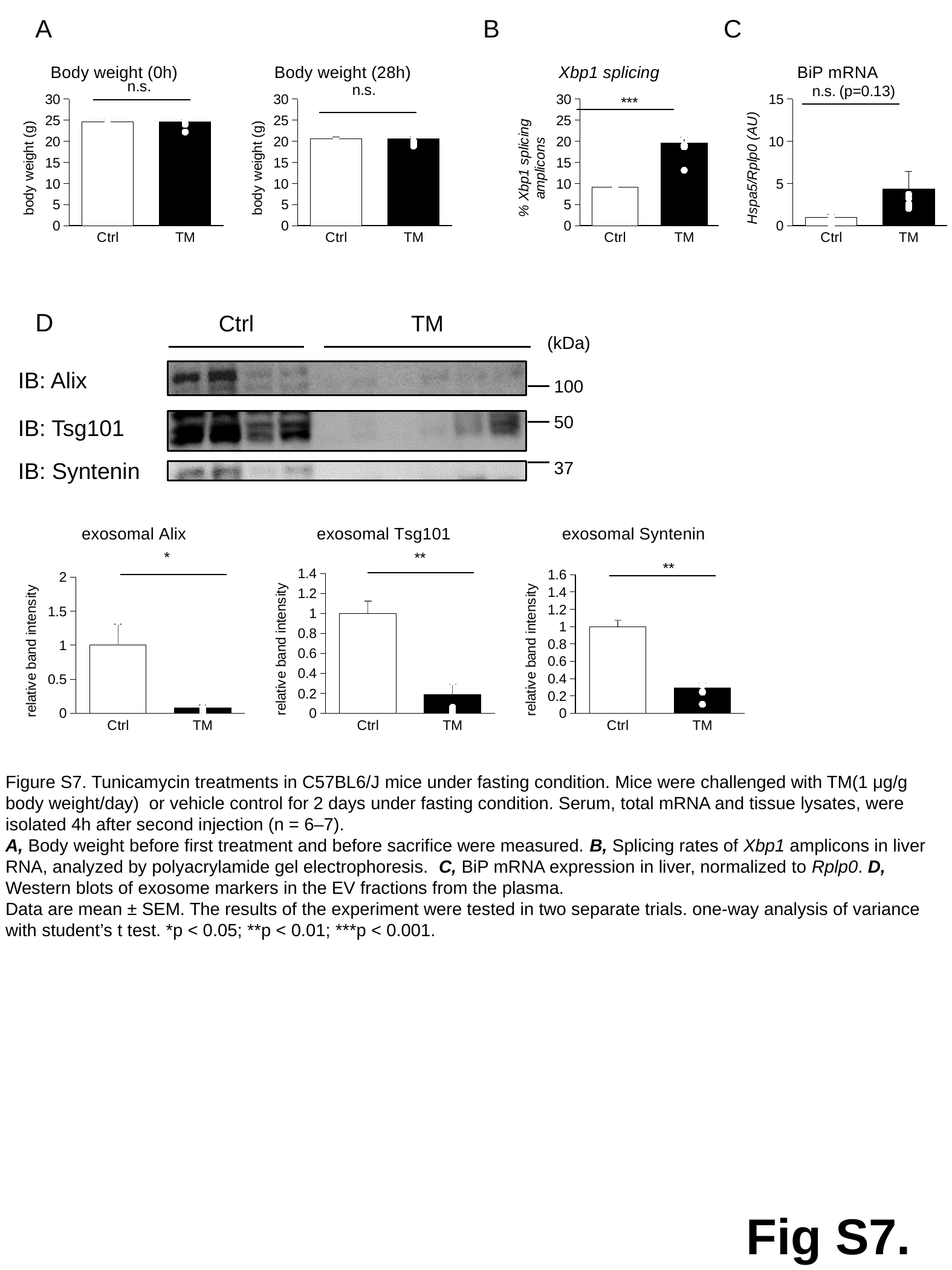

A
B
C
### Chart: Body weight (0h)
| Category | | | | | | | |
|---|---|---|---|---|---|---|---|
| Ctrl | 24.575 | 25.0 | 24.2 | 23.4 | 25.7 | None | None |
| TM | 24.633333333333336 | 24.2 | 22.2 | 24.0 | 26.4 | 25.6 | 25.4 |
### Chart: Body weight (28h)
| Category | | | | | | | |
|---|---|---|---|---|---|---|---|
| Ctrl | 20.6 | 20.2 | 20.2 | 20.1 | 21.9 | None | None |
| TM | 20.633333333333336 | 19.5 | 18.9 | 20.0 | 22.0 | 21.4 | 22.0 |
### Chart: Xbp1 splicing
| Category | | | | | | | |
|---|---|---|---|---|---|---|---|
| Ctrl | 9.150216683680958 | 9.265612284634278 | 9.30032734176832 | 9.226550978553973 | 8.808376129767263 | None | None |
| TM | 19.62827430643044 | 18.6838108111996 | 23.061687456203906 | 19.078167358203697 | 21.314595523134255 | 22.487450026367643 | 13.143934663473548 |
### Chart: BiP mRNA
| Category | | | | | | | |
|---|---|---|---|---|---|---|---|
| Ctrl | 1.0 | 0.9120508571673368 | 1.9129779062828591 | 1.067727904112848 | 0.10724333243695625 | None | None |
| TM | 4.367216216172151 | 12.17787338791036 | 3.2451576126472026 | 2.4399095610331463 | 2.580938406089925 | 3.716047379690278 | 2.0433709496619947 |D
Ctrl
TM
(kDa)
IB: Alix
100
50
IB: Tsg101
IB: Syntenin
37
### Chart: exosomal Alix
| Category | | | | | | | |
|---|---|---|---|---|---|---|---|
| Ctrl | 1.0 | 1.351019556714472 | 1.8330117340286831 | 0.451754889178618 | 0.36421382007822684 | None | None |
| TM | 0.07953585397653194 | 0.0037235984354628423 | 0.035963494132985656 | 0.0 | 0.08155410691003911 | 0.08113689700130378 | 0.27483702737940024 |
### Chart: exosomal Tsg101
| Category | | | | | | | |
|---|---|---|---|---|---|---|---|
| Ctrl | 1.0 | 1.179759218761607 | 1.2779953549974155 | 0.6565547829989696 | 0.8856906432420079 | None | None |
| TM | 0.18584902266257056 | 0.0077447769604144625 | 0.03174059216147402 | 0.0 | 0.059494650844427094 | 0.3158767410024105 | 0.7002373750066972 |
### Chart: exosomal Syntenin
| Category | | | | | | | |
|---|---|---|---|---|---|---|---|
| Ctrl | 1.0 | 1.2907110821376904 | 1.4835722661717694 | 0.48880901621532735 | 0.7369076354752131 | None | None |
| TM | 0.2926831019048719 | 0.10269903076076764 | 0.24803597402571978 | 0.24193589434881257 | 0.32438093783830874 | 0.4022489639065568 | 0.4367978105490659 |Figure S7. Tunicamycin treatments in C57BL6/J mice under fasting condition. Mice were challenged with TM(1 μg/g body weight/day) or vehicle control for 2 days under fasting condition. Serum, total mRNA and tissue lysates, were isolated 4h after second injection (n = 6–7).
A, Body weight before first treatment and before sacrifice were measured. B, Splicing rates of Xbp1 amplicons in liver RNA, analyzed by polyacrylamide gel electrophoresis. C, BiP mRNA expression in liver, normalized to Rplp0. D, Western blots of exosome markers in the EV fractions from the plasma.
Data are mean ± SEM. The results of the experiment were tested in two separate trials. one-way analysis of variance with student’s t test. *p < 0.05; **p < 0.01; ***p < 0.001.
Fig S7.
